# Association Between Ambient PM_2.5_ Exposure and Leukocyte Telomere Length Among Rural Adults: Cross-Sectional Study in Northern Thailand

**DOI:** 10.3390/ijms27219869

**Published:** 2026-11-04

**Authors:** Kanyapak Kohsuwan, Sayamon Hongjaisee, Kawinwut Somsunun, Nattawadee Promkam, Teetawat Santijitpakdee, Tippawan Prapamontol

**Affiliations:** 1 Research Institute for Health Sciences (RIHES), Chiang Mai University, Chiang Mai 50200, Thailandkawinwut.som@cmu.ac.th (K.S.); nattawadee_promkam@cmu.ac.th (N.P.);; 2 School of Health Sciences Research, Chiang Mai University, Chiang Mai 50200, Thailand; 3 Office of Research Administration, Chiang Mai University, Chiang Mai 50200, Thailand

**Keywords:** PM_2.5_, biomass burning, telomere dynamics, oxidative stress, aging biomarker, environmental health, Chiang Mai, Thailand

## Abstract

Ambient fine particulate matter (PM_2.5_) exposure has been associated with oxidative stress, inflammation, and biological aging; however, evidence regarding its relationship with leukocyte telomere length (LTL) remains inconsistent, particularly in rural Southeast Asian populations. This study aimed to investigate the association between ambient PM_2.5_ exposure and LTL across multiple exposure windows and examine whether oxidative stress and systemic inflammation were associated with PM_2.5_ exposure and LTL. This cross-sectional study included 179 adults from rural communities in Northern Thailand affected by biomass-burning pollution. Cumulative average ambient PM_2.5_ exposure was estimated for lag 0–7, 0–14, 0–30, 0–60, and 0–90 days, corresponding to 8, 15, 31, 61, and 91 days, respectively, including the blood-collection date (day 0). Relative LTL was quantified using quantitative polymerase chain reaction. Serum 8-hydroxy-2′-deoxyguanosine (8-OHdG), the Systemic Immune-Inflammation Index (SII), and the Systemic Inflammation Response Index (SIRI) were also assessed. Analyses included covariate-adjusted linear regression, exploratory wild cluster bootstrap inference, and sensitivity analyses. All *p*-values were unadjusted for multiple comparisons. Median relative LTL was 0.81 T/S units (Q1–Q3: 0.72–0.89). Adjusted models showed positive associations between PM_2.5_ and LTL at 0–30-, 0–60-, and 0–90-day windows, but not at 0–7- and 0–14-day windows. Per window-specific IQR increase in PM_2.5_ (8.13, 7.25, and 6.25 µg/m^3^, respectively), relative LTL was higher by 0.050 T/S units (95% CI: 0.012–0.089; nominal *p* = 0.011), 0.079 T/S units (95% CI: 0.035–0.123; nominal *p* < 0.001), and 0.094 T/S units (95% CI: 0.041–0.147; nominal *p* < 0.001). The reported confidence intervals and conventional *p*-values did not account for site–date clustering. In exploratory wild cluster bootstrap analyses using eight site-date groups, none of the five associations reached nominal significance (*p* = 0.247–0.787). The 0–30-day PM_2.5_ model explained an additional 3.8% of the variance in LTL (ΔR^2^ = 0.038; *p* = 0.011), while 8-OHdG contributed a further 2.4% (ΔR^2^ = 0.024; *p* = 0.041); SII and SIRI did not significantly improve model fit (*p* = 0.499 and 0.603, respectively). Sensitivity analyses showed positive longer-window associations with substantially varying magnitudes, whereas inverse shorter-window associations emerged in the NTAQHI-only analysis. Higher estimated ambient PM_2.5_ exposure was positively associated with higher measured LTL across the 0–30-, 0–60-, and 0–90-day windows in conventional adjusted models. However, sensitivity to sample composition and nonsignificant wild cluster bootstrap findings limit confidence in the robustness of these associations. Adding serum 8-OHdG, SII, or SIRI produced modest changes in model fit and did not establish mediation by oxidative stress or systemic inflammation. These exploratory findings should not be interpreted as evidence of within-person telomere change or a causal or protective effect of PM_2.5_.

## 1. Introduction

Ambient fine particulate matter (PM_2.5_; aerodynamic diameter ≤ 2.5 μm) is a major environmental risk factor and a key contributor to the global burden of disease. Exposure to PM_2.5_ has been associated with premature mortality and increased risks of cardiovascular, respiratory, metabolic, and malignant diseases worldwide [1,2]. More than 90% of the global population was estimated to live in areas where annual PM_2.5_ concentrations exceeded the 2005 World Health Organization (WHO) air quality guideline of 10 μg/m^3^, leading the WHO to establish a substantially lower annual guideline level of 5 μg/m^3^ in 2021 [3]. Recent global burden estimates further underscore the substantial health impact of PM_2.5_ exposure [4]. Beyond these established health effects, increasing evidence suggests that PM_2.5_ may contribute to biological aging through oxidative stress, chronic inflammation, immune dysregulation, mitochondrial dysfunction, and genomic instability [5].

PM_2.5_ exposure is a major concern in Southeast Asia, where recurrent seasonal haze is strongly driven by agricultural residue burning, forest fires, and transboundary smoke transport, particularly during the February–April dry season. Open biomass burning contributes substantially to PM_2.5_ concentrations and associated health burdens across mainland Southeast Asia [6]. In Northern Thailand, PM_2.5_ concentrations during the burning season have been reported to reach approximately 104 ± 45 μg/m^3^ in rural areas and 94 ± 39 μg/m^3^ in urban areas, substantially exceeding the WHO annual guideline level [7]. Northern Thailand is particularly vulnerable because of its extensive forest and agricultural areas, dry conditions, mountainous terrain, and regional transport of biomass-burning emissions. In Chiang Mai, mean PM_2.5_ concentrations have been reported to reach approximately 116 ± 35 μg/m^3^ during smoke-haze periods, with biomass burning contributing about 59 μg/m^3^ of PM_2.5_ [8]. Source-attribution studies indicate that both local and transboundary biomass burning contribute substantially to PM_2.5_ during the haze season, which accounts for a large proportion of the region’s annual PM_2.5_-attributable health burden [9,10]. Chiang Mai Province experiences recurrent, intense smoke-haze episodes, with substantially higher PM_2.5_ concentrations during burning periods, and biomass burning is a major source [8]. Rural communities may be particularly exposed because residential, agricultural, and occupational activities often occur near burning sources, with seasonal haze persisting for weeks to months and resulting in repeated exposure [11,12].

Telomeres are protective structures at the ends of chromosomes that progressively shorten with cell division and aging. Accelerated telomere shortening has been associated with increased risks of cardiovascular disease, diabetes, chronic obstructive pulmonary disease, cancer, and other age-related conditions [13,14,15]. Because telomeres are particularly vulnerable to oxidative damage and inflammatory stress, leukocyte telomere length (LTL) has increasingly been investigated as a potential molecular marker linking environmental exposures to biological aging [16,17]. Ambient PM_2.5_ may influence telomere dynamics through several interconnected biological pathways. PM_2.5_ can generate reactive oxygen species and induce oxidative stress, systemic inflammation, mitochondrial dysfunction, and DNA damage, which may increase telomere attrition and impair telomere maintenance [18]. However, the relationship between ambient PM_2.5_ exposure and LTL remains inconsistent. Several epidemiological studies have reported that higher long-term PM_2.5_ exposure is associated with shorter LTL, supporting the hypothesis that chronic particulate exposure accelerates telomere attrition and biological aging [19,20]. A recent meta-analysis including 20 studies and approximately 483,600 participants found that higher long-term PM_2.5_ exposure was associated with shorter telomere length, with stronger associations reported among Asian populations [20].

In contrast, other studies have reported null or positive associations, particularly for short-term or intermediate-term exposure [21,22,23]. For example, a repeated-measures study in highly exposed workers in Beijing reported a positive association between same-day PM_2.5_ exposure and LTL, whereas longer-term exposure was associated with shorter telomeres [22]. Although telomere shortening may represent the predominant response to sustained oxidative and inflammatory stress, positive associations may reflect differences in exposure timing, cell composition, telomerase activation, cellular turnover, or compensatory responses to acute or intermittent stress [19]. Acute exposure may alter the distribution of leukocyte subsets with inherently different telomere lengths, while transient telomerase activation or selective expansion of leukocyte populations with relatively longer telomeres could also contribute to an apparent increase in measured LTL [24]. These mechanisms provide plausible explanations for why telomere lengthening may be observed under some exposure conditions and highlight that the relationship between PM_2.5_ exposure and LTL may not be uniformly unidirectional but may depend on the timing, duration, intensity, and biological response to exposure [20,23,25,26].

Several biological mechanisms may link PM_2.5_ exposure to telomere length. Redox-active components of fine particles can generate reactive oxygen species (ROS) and activate inflammatory pathways, causing oxidative DNA damage, particularly in guanine-rich telomeric regions [27,28]. Chronic inflammation may also increase leukocyte turnover and impair telomere maintenance by affecting telomerase activity and telomere-associated proteins [29]. However, environmental stress may produce more complex telomere responses. Changes in leukocyte composition, cellular turnover, and compensatory telomere-maintenance mechanisms may contribute to higher measured LTL under some exposure conditions [29,30,31,32]. Thus, a positive association with PM_2.5_ may reflect the balance between telomere damage, cellular maintenance, and leukocyte composition rather than direct telomere elongation. Serum 8-hydroxy-2′-deoxyguanosine (8-OHdG) and complete blood count-derived indices, including the systemic immune-inflammation index (SII) and systemic inflammation response index (SIRI), measure oxidative DNA damage and systemic inflammation, respectively [33,34,35,36,37]. However, their potential roles in the association between PM_2.5_ exposure and LTL remain poorly understood, particularly in populations exposed to recurrent seasonal biomass-burning pollution.

Northern Thailand experiences recurrent seasonal smoke haze caused primarily by biomass burning during the dry season [10], with PM_2.5_ concentrations that frequently exceed both Thailand’s National Ambient Air Quality Standards of 37.5 µg/m^3^ for the 24-h average [38] and the WHO Air Quality Guideline levels of 15 µg/m^3^ for the 24-h average [3]. Rural communities in Chiang Mai Province, including Omkoi and Mae Chaem, are particularly affected by seasonal biomass-burning pollution. Unlike continuous urban exposure, biomass-burning episodes show substantial temporal variation, with repeated periods of elevated PM_2.5_ exposure lasting from weeks to months [39,40,41].

Accurately characterizing PM_2.5_ exposure remains challenging in rural and mountainous settings, where conventional air-quality monitoring stations are limited and fixed-site measurements may not adequately reflect local spatial variability in pollution [42,43]. In Northern Thailand, the Northern Thailand Air Quality and Health Index (NTAQHI) network provides localized PM_2.5_ data using low-cost optical sensors. Because these sensors can be affected by humidity and degrade over time, they require appropriate calibration and correction procedures. After correction, field testing in Chiang Mai showed strong agreement between NTAQHI sensor measurements and reference-monitor data (R^2^ = 0.9737) [44]. Meanwhile, the Geo-Informatics and Space Technology Development Agency (GISTDA) provides broader spatial PM_2.5_ information derived from satellite observations. Integrating quality-controlled NTAQHI measurements with GISTDA-derived spatial estimates and data from conventional reference stations may therefore improve the accuracy and spatial representativeness of PM_2.5_ exposure estimates for rural communities affected by seasonal biomass-burning haze.

Therefore, this study aimed to (1) investigate the association between ambient PM_2.5_ exposure and relative LTL across multiple exposure windows among adults living in biomass-burning-affected rural communities in Northern Thailand; (2) examine the associations of PM_2.5_ exposure with serum 8-OHdG and the systemic inflammatory indices SII and SIRI; and (3) examine whether the association between PM_2.5_ exposure and LTL changed after including these oxidative-stress and inflammatory biomarkers in the models. We hypothesized that higher ambient PM_2.5_ exposure would be associated with altered LTL and increased oxidative stress and systemic inflammation, and that including these biological factors might change the estimated PM_2.5_–LTL association. By integrating localized estimates with molecular and inflammatory biomarkers, this study provides evidence from a rural Southeast Asian population and contributes to a better understanding of the heterogeneous relationship between seasonal biomass-burning exposure and telomere biology.

## 2. Results

### 2.1. Participant Characteristics

Of the 194 individuals assessed for eligibility, 15 were excluded: 11 for incomplete study data or biological-sample collection and 4 for unstable noncommunicable diseases. The final analysis included 179 rural adults with complete PM_2.5_ exposure, covariate, and relative leukocyte telomere length data. Participants were distributed across eight monitoring site–date exposure groups at four study sites, with a median of 24 participants per group (range: 11–32). Participants within each group shared identical PM_2.5_ estimates for the corresponding exposure windows.

The study population comprised 50 males (27.9%) and 129 females (72.1%). Table 1 summarizes the participants’ demographic characteristics, lifestyle factors, inflammatory and oxidative-stress biomarkers, and relative LTL. The median age was 44.0 years (Q1, Q3: 36.0, 51.0), and the median duration of residence was 37.0 years (Q1, Q3: 24.5, 48.0); neither variable differed significantly by sex. BMI was significantly higher among females than males (25.4 vs. 23.8 kg/m^2^; *p* = 0.003). Smoking status and alcohol consumption also differed significantly by sex, with males more likely to be former or current smokers and current alcohol consumers (both *p* < 0.01). Median SIRI was higher among males than females (692.0 vs. 529.0 cells/mm^3^; nominal *p* = 0.034). No statistically significant sex differences were observed in CRP, SII, serum 8-OHdG, or relative LTL.

### 2.2. Correlations Between PM_2.5_ Exposure and Biomarker Parameters

Spearman’s rank correlation analysis was performed to investigate the correlation between clinical and laboratory parameters and PM_2.5_ exposure across five lag windows (Table 2). In the 0–30-day (r_s_ = 0.227, *p* = 0.002), 0–60-day (r_s_ = 0.253, *p* = 0.001), and 0–90-day (r_s_ = 0.243, *p* = 0.001) windows, LTL was positively correlated with PM_2.5_ exposure. However, no significant correlation was observed in the lag 0–7-day or lag 0–14-day windows. In contrast, the 0–60-day (r_s_ = −0.174, *p* = 0.020) and 0–90-day (r_s_ = −0.191, *p* = 0.011) windows revealed inverse correlations between PM_2.5_ exposure and SII. Furthermore, a negative correlation was observed between SIRI and PM_2.5_ exposure in the 0–30-day (r_s_ = −0.189, *p* = 0.011), 0–60-day (r_s_ = −0.220, *p* = 0.003), and 0–90-day (r_s_ = −0.204, *p* = 0.006) windows. Across all exposure windows, we observed no statistically significant correlations between PM_2.5_ exposure and either CRP or 8-OHdG.

### 2.3. Association Between Oxidative Stress and Leukocyte Telomere Length

**Figure 1:**
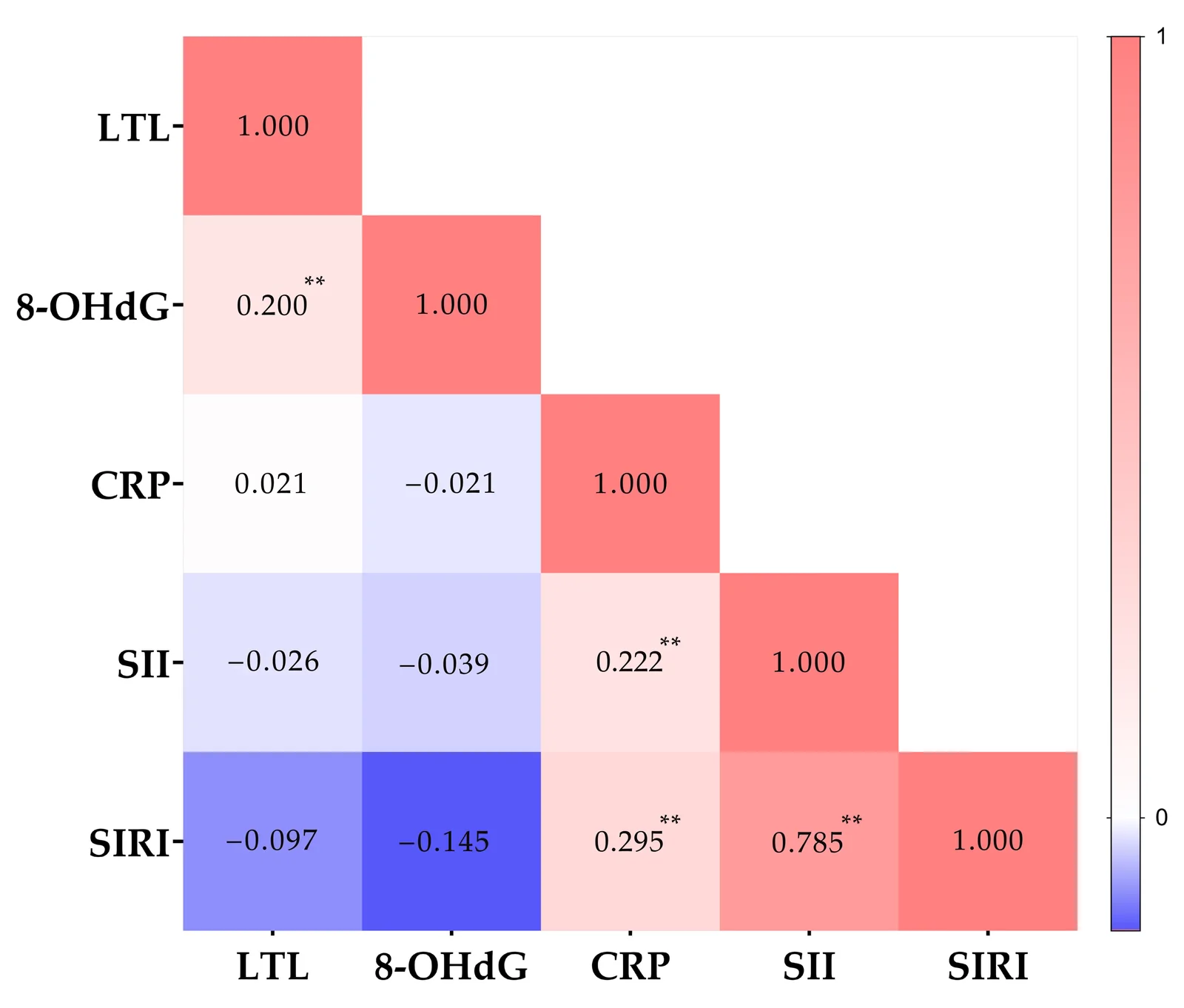
Spearman correlation heatmap among clinical and laboratory parameters. Blue indicates negative correlations, red indicates positive correlations, and white represents correlations close to zero. Color intensity corresponds to the magnitude of the correlation coefficient. ** *p* < 0.01 (Spearman’s rank correlation, two-tailed).

Spearman’s rank correlation analysis revealed a significant positive correlation between LTL and 8-OHdG (r_s_ = 0.200, *p* = 0.007). Among the inflammation-related biomarkers, CRP was positively correlated with the SII (r_s_ = 0.222, *p* = 0.003) and SIRI (r_s_ = 0.295, *p* < 0.001). The strongest correlation was observed between SII and SIRI (r_s_ = 0.785, *p* < 0.001). No significant correlations were found between LTL and the inflammation-related biomarkers or between 8-OHdG and any of the inflammation-related biomarkers (all *p* > 0.05). Figure 1 presents the correlation.

### 2.4. Multivariable Linear Regression Analysis of PM_2.5_ Exposure and Leukocyte Telomere Length

Multivariable linear regression models were fitted to assess the association between PM_2.5_ exposure across the five averaging periods and relative LTL among 179 participants (Table 3). All models were adjusted for sex, age, body mass index, smoking status, alcohol consumption, biomass-burning exposure, forest-fire exposure, education, occupation, and chronic disease and/or regular medication use. None of the adjustment covariates showed statistically significant associations with LTL in these models. After adjustment for the specified covariates, PM_2.5_ exposure was not significantly associated with relative LTL in the lag 0–7 or lag 0–14 windows. Each 10 µg/m^3^ increase in PM_2.5_ was associated with 0.062, 0.108, and 0.151 T/S units higher relative LTL in the lag 0–30, lag 0–60, and lag 0–90 windows, respectively. The corresponding estimates per window-specific IQR increase in PM_2.5_ (8.13, 7.25, and 6.25 µg/m^3^, respectively) were 0.050, 0.079, and 0.094 T/S units (Table 3). These lag windows comprised 31, 61, and 91 consecutive days, respectively, ending on and including the blood-collection date (day 0). These cross-sectional associations should not be interpreted as within-person telomere lengthening.

Regression diagnostics for the five exposure-window models are summarized in Supplementary Table S9. The Breusch–Pagan tests did not detect evidence of heteroskedasticity (*p* = 0.950–0.999). The Shapiro–Wilk tests indicated departures from residual normality in the 0–7- and 0–14-day models (*p* = 0.037 and 0.029, respectively), whereas the corresponding *p*-values for the 0–30-, 0–60-, and 0–90-day models were 0.097, 0.077, and 0.059. Across models, studentized residuals ranged from −2.79 to 3.33, maximum Cook’s distances ranged from 0.082 to 0.100, and maximum leverage values ranged from 0.162 to 0.191.

Exploratory wild cluster bootstrap analyses were conducted for the five exposure windows, with categorical covariates represented by indicator variables. Bootstrap *p*-values were 0.787, 0.554, 0.342, 0.247, and 0.321 for the 0–7-, 0–14-, 0–30-, 0–60-, and 0–90-day windows, respectively (Supplementary Table S8). Although the longer-window coefficients were positive, none of the five associations reached the nominal 0.05 significance threshold. In exploratory age-stratified analyses, each window-specific IQR increase in PM_2.5_ was positively associated with relative LTL among participants aged <35 years (*n* = 37) for the 0–30-, 0–60-, and 0–90-day windows (B = 0.271, 0.256, and 0.244 T/S units, respectively; all nominal *p* < 0.001). No statistically significant associations were observed among participants aged 35–49 years (*n* = 91) or ≥50 years (*n* = 51) (Table S6). Sensitivity analyses yielded larger positive coefficients after excluding Omkoi participants (*n* = 152): B = 0.146, 0.146, and 0.159 T/S units per IQR increase for the 0–30-, 0–60-, and 0–90-day windows, respectively. Corresponding coefficients in the NTAQHI-only analysis (*n* = 91) were 0.202, 0.170, and 0.173 (all nominal *p* < 0.001; Table S7). Additional adjustment for neutrophil and lymphocyte percentages yielded coefficients of 0.048, 0.075, and 0.090 for these windows (*p* = 0.016, 0.001, and 0.001, respectively); the 0–7- and 0–14-day associations remained nonsignificant. In the NTAQHI-only analysis, inverse associations were observed for the 0–7-day window (B = −0.099 per IQR; 95% CI: −0.189 to −0.008; *p* = 0.036) and the 0–14-day window (B = −0.283; 95% CI: −0.418 to −0.148; *p* < 0.001). These subgroup and sensitivity results were based on conventional regression inference without adjustment for site-date clustering or multiple comparisons.

Exploratory hierarchical linear regression models examined the incremental contributions of 0–30-day PM_2.5_ exposure and, separately, serum 8-OHdG, SII, and SIRI to relative LTL (Table 4). The covariate-only model explained 1.8% of LTL variability (R^2^ = 0.018; adjusted R^2^ = −0.041; overall *p* = 0.980). Adding PM_2.5_ increased the explained variance by 3.8 percentage points (ΔR^2^ = 0.038; F-change = 6.637; nominal *p* = 0.011). Each IQR increase in PM_2.5_ (8.13 µg/m^3^) was associated with 0.050 T/S units higher relative LTL (95% CI: 0.012–0.089; standardized β = 0.218), equivalent to 0.062 T/S units per 10 µg/m^3^. Adding serum 8-OHdG explained an additional 2.4 percentage points of variance (ΔR^2^ = 0.024; F-change = 4.246; nominal *p* = 0.041). Serum 8-OHdG was positively associated with relative LTL (B = 0.004 T/S units per ng/mL; 95% CI: 0.000–0.008; standardized β = 0.155). The PM_2.5_ coefficient was 0.047 T/S units per IQR (95% CI: 0.008–0.085; nominal *p* = 0.017). Adding SII or SIRI separately did not significantly improve model fit (ΔR^2^ = 0.003, *p* = 0.499; ΔR^2^ = 0.002, *p* = 0.603). The corresponding PM_2.5_ coefficients were 0.049 and 0.050 T/S units per IQR increase in the SII- and SIRI-adjusted models, respectively (SII-adjusted: 95% CI: 0.011–0.088, nominal *p* = 0.013; SIRI-adjusted: 95% CI: 0.011–0.089, nominal *p* = 0.012). Across these models, the PM_2.5_ coefficients ranged from 0.047 to 0.050 T/S units per IQR.

## 3. Discussion

This study examined associations between ambient PM_2.5_ exposure and oxidative DNA damage, inflammatory biomarkers, and LTL among adults living in rural communities affected by seasonal biomass-burning pollution in Northern Thailand. PM_2.5_ concentrations averaged across the lag 0–30, lag 0–60, and lag 0–90 exposure windows, corresponding to 31, 61, and 91 consecutive days ending on and including the blood-collection date (day 0), were positively associated with LTL after covariate adjustment, whereas the 0–7- and 0–14-day exposure windows showed no significant associations. For each window-specific IQR increase in PM_2.5_ (8.13, 7.25, and 6.25 µg/m^3^, respectively), relative LTL was higher by 0.050 T/S units for the 0–30-day window, 0.079 T/S units for the 0–60-day window, and 0.094 T/S units for the 0–90-day window. Positive associations were most evident for the longer moving-average exposure windows in conventional adjusted models. However, none reached nominal significance in the exploratory wild cluster bootstrap analyses, limiting confidence in the statistical evidence for these associations. Because these exposure windows overlap and are likely strongly correlated, the progressively larger estimates across longer windows should not be interpreted as independent cumulative effects or as evidence of a definitive exposure–response relationship.

Relative LTL had a median of 0.81 T/S units (Q1–Q3: 0.72–0.89) and a mean of 0.81 ± 0.15 T/S units. These values were numerically lower than reported for several Thai groups. Autsavapromporn et al. (2018) reported a mean T/S ratio of 1.12 ± 0.30 among healthy nonsmokers in Chiang Mai [45]. Winson et al. (2022) reported values of 1.08 ± 0.18 among Thai Buddhist monks and 1.02 ± 0.17 among Thai men aged ≥40 years [46], while Nantanawut et al. (2022) reported age-specific means ranging from 0.96 to 1.19 in normal-weight Thai men and from 0.90 to 1.12 in men with obesity [47]. These differences in T/S ratios should be interpreted cautiously because they may reflect methodological variation between assays and laboratories [48,49], as well as population-specific and environmental factors. In particular, participants in the present study lived in rural communities in Northern Thailand that experience recurrent seasonal biomass burning and PM_2.5_ exposure, which may represent an additional environmental stressor that could affect telomere biology. However, the present cross-sectional study cannot determine whether repeated or cumulative exposure to air pollution contributed to the lower LTL observed in this population. Other unmeasured factors, including lifestyle, occupational exposures, socioeconomic conditions, and genetic or biological differences, may also have contributed. Thus, the reasons for the relatively lower T/S ratios observed in this population remain uncertain and warrant further investigation using standardized telomere measurement protocols and longitudinal exposure assessment.

In multivariable linear regression models, the positive associations between PM_2.5_ and LTL at 30–90 days differ from the commonly proposed model of cumulative pollution-related telomere shortening. Nevertheless, positive associations with particulate exposure have been reported. Dioni et al. (2011) observed an increase in mean LTL from 1.23 T/S units (95% CI: 1.16–1.30) to 1.43 T/S units (95% CI: 1.29–1.57) after three consecutive workdays [26]. Hou et al. (2012) found that an interquartile-range increase in personal PM_2.5_ exposure was associated with 5.2% longer LTL [22]. Pieters et al. (2016) similarly reported longer LTL in association with preceding-month PM_2.5_ exposure, although annual exposure was associated with a 16.8% reduction in LTL per 5 µg/m^3^ increase [50]. Hautekiet et al. (2025) also observed 2.4% longer LTL per interquartile-range increase in one-week PM_2.5_ exposure [21]. Conversely, cumulative welding-fume PM_2.5_ exposure and household biomass-smoke exposure have been associated with shorter telomeres [29,51]. Large population studies generally support inverse associations between chronic air pollution and LTL, although results vary by pollutant, population, biological specimen, exposure window, and analytical approach [52,53]. These findings therefore add to evidence that the association between PM_2.5_ and LTL may differ by exposure context and time window, rather than following a uniform direction. Although the associations persisted after additional adjustment for neutrophil and lymphocyte percentages, residual confounding related to leukocyte composition cannot be excluded. Leukocyte subpopulations may differ in telomere length, and adjusting for differential leukocyte percentages may not fully account for variation in blood sample cellular composition.

Age-stratified analyses provided additional information on the observed association. Among participants aged <35 years, PM_2.5_ exposure was positively associated with relative LTL for the 0–30-, 0–60-, and 0–90-day exposure windows (B = 0.271, 0.256, and 0.244 T/S units per IQR increase in PM_2.5_, respectively). No statistically significant associations were observed among participants aged 35–49 or ≥50 years. These exploratory age-stratified findings should be interpreted cautiously. Nominally significant associations among participants aged <35 years, alongside nonsignificant findings in older age groups, do not establish differences in the associations between age groups. Limited subgroup sample sizes and the limited number of shared site–date exposure groups limit the precision and stability of the estimates. Without a formal PM_2.5_-by-age interaction test, these findings remain hypothesis-generating and do not establish effect modification by age.

Several biological mechanisms may potentially explain the positive association observed in this study. PM_2.5_ exposure may induce cellular stress and inflammatory responses that activate compensatory pathways involved in cell survival and telomere maintenance. In addition, changes in circulating leukocyte composition may influence measured relative LTL because different leukocyte subsets have distinct telomere lengths. Short-term or intermediate-term exposure may therefore change circulating cell populations or cellular turnover in ways that affect the measured T/S ratio without reflecting true telomere elongation. Telomerase activation and other cellular stress-response mechanisms may also contribute to transient preservation of telomere length under repeated environmental stress. These mechanisms remain speculative in the present study because telomerase activity, leukocyte-subset-specific telomere length, and longitudinal changes in LTL were not measured.

Although serum 8-OHdG was positively associated with LTL and contributed modestly to the variance explained by the 0–30-day model, PM_2.5_ was not significantly associated with serum 8-OHdG at any examined exposure window. These cross-sectional analyses were not designed to establish mediation. The inclusion of serum 8-OHdG, SII, or SIRI separately resulted in limited attenuation of the PM_2.5_ coefficient and modest changes in model fit. However, these exploratory cross-sectional model comparisons cannot establish mediation or rule out potential contributions of oxidative stress and systemic inflammation to the observed association. Previous studies have reported higher urinary 8-OHdG with ambient PM_2.5_ and biomass-smoke exposure [35,54]. Differences may reflect the biological matrix, sampling time, particle composition, assay methodology, and intra-individual variability. Serum 8-OHdG may represent a combination of oxidative DNA modification, DNA repair, and underlying disease processes and should not be interpreted as a direct measure of PM_2.5_-induced DNA damage.

The inflammatory indices provided limited explanatory information in the fitted models. Although exploratory correlations were observed between longer PM_2.5_ exposure windows and the SII or SIRI, adding either index separately produced little change in the estimated PM_2.5_ coefficient and did not significantly improve model fit (SII: ΔR^2^ = 0.003; F-change = 0.458; *p* = 0.499; SIRI: ΔR^2^ = 0.002; F-change = 0.271; *p* = 0.603). These exploratory model comparisons do not establish mediation or exclude the contribution of systemic inflammation. These indices reflect broad changes in circulating neutrophils, lymphocytes, monocytes, and platelets but may not capture the specific cytokine signaling or intracellular responses involved in telomere regulation [18,55]. Future studies should incorporate high-sensitivity CRP, IL-6, TNF-α, additional redox biomarkers, telomerase activity, and leukocyte-subset-specific telomere measurements [18,29].

Sensitivity analyses showed positive associations for the longer exposure windows, but the magnitude of the estimates varied substantially across analyses. Excluding Omkoi increased the 0–30-day coefficient from 0.050 to 0.146 T/S units per 8.13 µg/m^3^ increase in PM_2.5_, corresponding to the IQR among all 179 participants. After excluding Omkoi, the coefficient was approximately 2.9 times the estimate among all participants. This change indicates sensitivity to sample composition, despite the association remaining in the same direction. Accordingly, these analyses provide supplementary information but do not establish the robustness of the estimated effects. In the NTAQHI-only analysis, inverse associations were observed for the 0–7- and 0–14-day windows, whereas positive associations were observed for the 0–30-, 0–60-, and 0–90-day windows. This analysis included 91 participants from two monitoring sites and four site–date exposure groups, with participants within each group assigned the same exposure estimate. Consequently, the exposure coefficients were estimated from contrasts between only four groups and may reflect between-group differences in LTL and other site–date characteristics. Differences in the ordering and spacing of group-level exposure estimates across windows may also contribute to changes in the direction and magnitude of the coefficients. The identical median 0–7-day PM_2.5_ concentration among all participants and among those included in the NTAQHI-only analysis (58.38 µg/m^3^) does not imply identical exposure distributions or exposure–LTL associations. Furthermore, restricting to NTAQHI-only data changed the participants and sites included; therefore, differences from the analysis among all participants cannot be attributed solely to the exposure data source. These findings should be regarded as exploratory and potentially unstable and should not be interpreted as evidence of within-person telomere shortening or lengthening.

Several limitations should be acknowledged. First, the cross-sectional design and single LTL measurement preclude causal inference and assessment of within-person telomere change. Residual analytical batch effects cannot be excluded. Although samples were randomly distributed across four batches and normalized using a common reference DNA sample, the inter-assay CV for the T/S ratio was 9.56%. Batch was not included as a covariate, and no batch-adjusted sensitivity analysis was performed. Given the modest magnitude of the estimated associations, between-batch measurement variability may affect their precision and, if associated with exposure assignment, could influence the estimates. The direction and magnitude of any such influence remain uncertain. Second, the low or negative adjusted R^2^ values indicate that the fitted models explained little LTL variability after accounting for model complexity. None of the adjustment covariates, including age, showed statistically significant associations with LTL; however, this does not rule out such associations. The reasons for these nonsignificant findings remain uncertain. Unmeasured genetic, behavioral, and environmental determinants may contribute to unexplained LTL variability and, where associated with PM_2.5_ exposure, to residual confounding, although low model fit alone does not demonstrate such confounding. Ozone, nitrogen dioxide, and agricultural chemical exposures, including pesticides, were not measured. These co-exposures may vary by site, season, or occupation and could contribute to residual confounding of the observed PM_2.5_ –LTL associations. Third, the five overlapping exposure windows were likely correlated, limiting interpretation of the progressively larger estimates across longer windows as evidence of an exposure–response relationship. Analyses across exposure windows, biomarkers, and age strata were not adjusted for multiple comparisons. Consequently, nominally significant findings exploratory and requiring confirmation in independent studies. PM_2.5_ exposure was assigned to 179 participants across only eight site–date groups. Conventional OLS confidence intervals and *p*-values did not account for within-group dependence and may overstate precision. Exploratory wild cluster bootstrap analyses addressed the specified site–date clustering structure, but inference remained limited by the small number of clusters and potential dependence between groups sharing a monitoring site. Fourth, PM_2.5_ exposure was estimated at the community/site level rather than directly measured for each participant. Individual exposure may have varied according to occupation, household fuel use, indoor infiltration, time–activity patterns, and protective behaviors. The observed associations therefore reflect estimated ambient PM_2.5_ concentrations rather than individual exposure doses. GISTDA data were used to fill missing NTAQHI observations and support the calculation of retrospective PM_2.5_ averages. Although supplementation improved daily data coverage, it did not align the two sources. Comparisons of paired raw GISTDA estimates and NTAQHI measurements yielded slopes of 1.017–1.719 and intercepts of −11.580 to 35.340 µg/m^3^ (Supplementary Table S5), suggesting potential proportional and additive differences. These comparisons describe the uncalibrated data and should be distinguished from the SVR-calibrated estimates used for supplementation. However, evidence for these differences varied by site: slope confidence intervals included 1 at Omkoi and Mae Na John, and confidence intervals for the intercepts were not reported. Strong linear associations alone also do not demonstrate that the two sources are interchangeable. Differences between NTAQHI and GISTDA values may therefore have affected the exposure-window averages and the estimated PM_2.5_–LTL associations. The magnitude and direction of this potential influence remain uncertain. In addition, estimated ambient PM2.5 concentrations may not fully reflect individual-level exposure, which could lead to exposure misclassification. Fifth, the predominance of women limits generalizability to men and populations with different sex distributions. Adjustment for sex does not remove potential selection bias or establish that the PM_2.5_–LTL association is equivalent in men and women. Finally, monitoring site and blood-collection date may confound the observed associations because they may be associated with both PM_2.5_ concentrations and participant characteristics. The limited number of site–date groups restricts the ability to distinguish PM_2.5_ associations from spatial and temporal differences.

This study has several strengths, including its focus on an understudied rural population experiencing substantial seasonal exposure to biomass-burning-related PM_2.5_ in Northern Thailand, its examination of multiple cumulative exposure windows, and its integration of ambient air pollution estimates with telomere, oxidative-stress, and hematological measurements. By examining exposure across time scales, the study offers a more nuanced view of the temporal patterns of PM_2.5_ exposure in relation to measured relative LTL. These findings add to the limited evidence on potential biological responses to seasonal air pollution in rural communities of Northern Thailand and highlight the importance of sustained efforts to reduce biomass burning and ambient PM_2.5_ exposure. Such efforts may be particularly relevant during the burning season, when rural communities can experience PM_2.5_ concentrations that exceed the World Health Organization air quality guideline levels. Although the positive associations at the 0–30-, 0–60-, and 0–90-day exposure windows persisted after additional adjustment for neutrophil and lymphocyte percentages, differential leukocyte composition may not have been fully captured. Therefore, residual cell-composition-related bias cannot be excluded, and these findings should be interpreted cautiously.

## 4. Materials and Methods

### 4.1. Study Design and Participants

**Figure 2:**
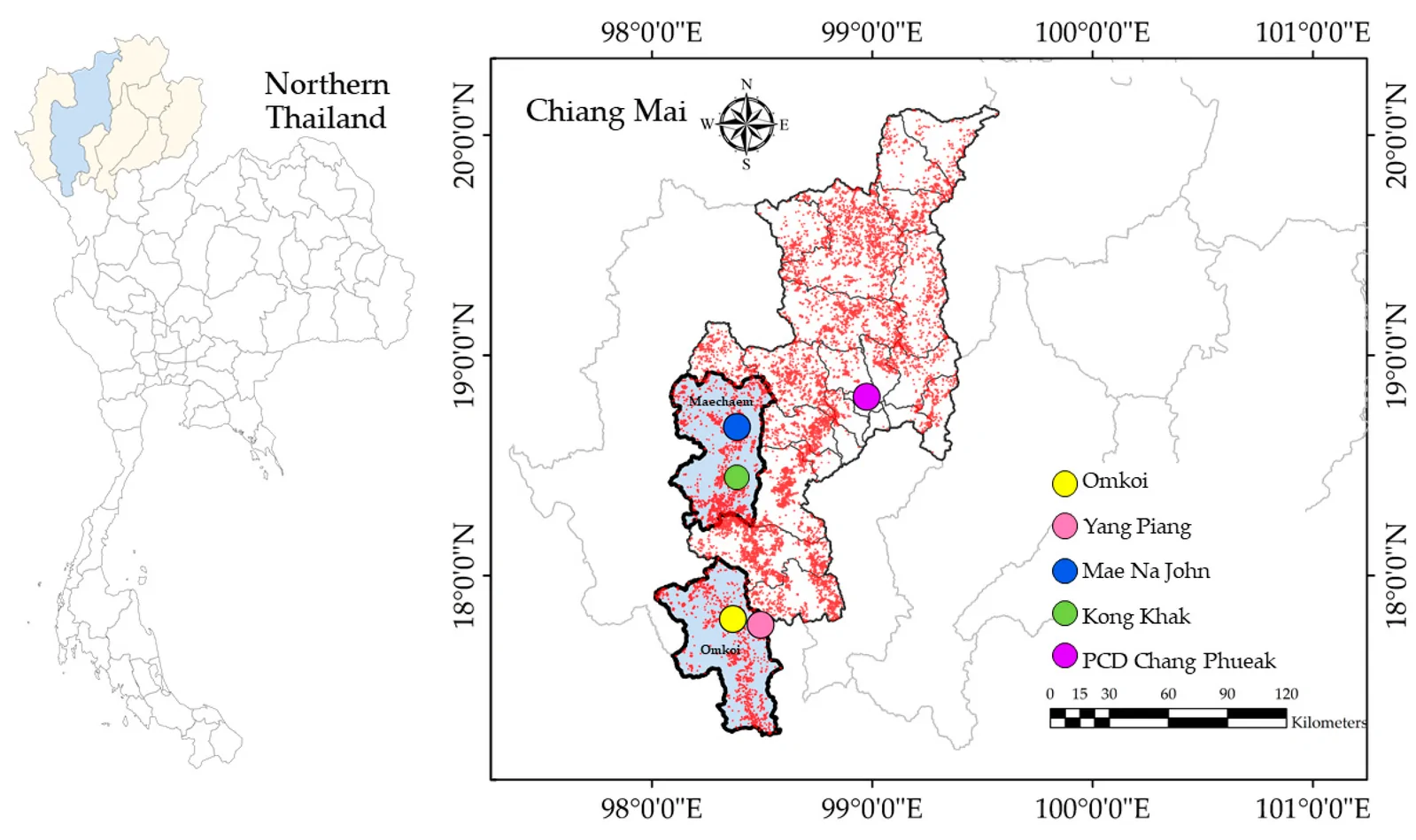
Geographic locations of the study sites and air-quality monitoring stations in Chiang Mai Province, Northern Thailand. Study sites were located in Omkoi (17°48′ N, 98°21′ E) and Mae Chaem (19°07′ N, 98°34′ E) districts. Colored circles show the study sites and monitoring stations, including NTAQHI and the main PCD monitoring station in Chiang Mai city. Yellow shading indicates the upper northern region of Thailand, while blue shading highlights Chiang Mai Province, where the study was conducted. Red dots indicate fire hotspots detected from January to April 2024.

This cross-sectional study included adults residing in Omkoi and Mae Chaem districts, Chiang Mai Province, Thailand, areas characterized by high biomass burning and frequent PM_2.5_ concentrations exceeding WHO air quality guidelines (Figure 2). We collected data across eight site–date combinations from four monitoring sites in the Omkoi and Mae Chaem districts of Chiang Mai Province between March and April 2024. The study protocol was approved by the Ethics Committee of the Research Institute for Health Sciences (Approval No. 15/2024), and all participants provided written informed consent before enrollment.

Participants were recruited through community-based volunteer recruitment in selected rural communities in the study area. Approximately 50 individuals were invited from each selected village. Participants were eligible if they were aged 18–60 years and had lived continuously in the study area for at least 5 years before recruitment. Participants were excluded if they had a history of unstable, non-communicable diseases (e.g., uncontrolled diabetes mellitus or exacerbated chronic obstructive pulmonary disease) within the past year and had incomplete study data or incomplete biological sample collection. An a priori sample-size calculation was performed using G*Power 3.1 for a fixed multiple-regression model testing a single regression coefficient, with relative LTL as the outcome and PM_2.5_ exposure as the primary predictor. The model included 10 predictors. Assuming a medium effect size (f^2^ = 0.15), a two-sided significance level of (α = 0.05), and 95% statistical power, the minimum required sample size was estimated at 89 participants. After allowing for 20% incomplete observations, the target sample size was increased to 107 participants. A total of 194 individuals were assessed for eligibility, of whom 179 were included in the primary analysis after applying the predefined eligibility or data-completeness criteria. The a priori sample-size calculation did not account for dependence among participants sharing the same site–date exposure assignment. The study is reported in accordance with the STROBE guidelines for cross-sectional studies, and the participant flow diagram is provided in Figure S1.

All participants were interviewed by trained researchers using a structured questionnaire. Information on demographic characteristics (age, sex, and education level), lifestyle factors (smoking status and alcohol consumption), and occupational and environmental exposure history was collected. Anthropometric measurements, including body weight and height, were obtained to calculate body mass index (BMI). Following an overnight fast, 15 mL of venous blood was collected from each participant. The blood-collection date was defined as day 0 and served as the endpoint for all cumulative exposure windows (lag 0–7, 0–14, 0–30, 0–60, and 0–90 days). Blood samples were analyzed for complete blood count (CBC), including WBC count, differential leukocyte percentages, and platelet count; these measurements were used to calculate the systemic immune-inflammation index (SII) and systemic inflammation response index (SIRI). Serum samples were also analyzed for C-reactive protein (CRP) and 8-hydroxy-2′-deoxyguanosine (8-OHdG), while leukocyte telomere length was determined from genomic DNA extracted from the buffy coat. CBC and CRP analyses were performed immediately after sample collection. Serum samples designated for 8-OHdG analysis were separated, aliquoted, and stored at −20 °C for approximately 2 months before analysis. Buffy-coat fractions were isolated, aliquoted, and stored at −80 °C for approximately 2 months before genomic DNA extraction for leukocyte telomere-length analysis.

### 4.2. Clinical Laboratory Analysis

Complete blood count (CBC) parameters were measured using an automated hematology analyzer (XN-1000, Sysmex Corporation, Kobe, Japan). White blood cell (WBC) and platelet counts were expressed as cells/mm^3^, while differential leukocyte counts, including neutrophils, lymphocytes, and monocytes, were reported as percentages (%). Absolute neutrophil, lymphocyte, and monocyte counts were derived by multiplying the WBC count by the corresponding differential percentage and dividing by 100. SII and SIRI were calculated using platelet counts and absolute leukocyte counts, rather than differential percentages, as follows:

(1) SII = (platelets × neutrophils)/lymphocytes

and the Systemic Inflammation Response Index

(2) SIRI = (neutrophils × monocytes)/lymphocytes

Both indices are reported in cells/mm^3^. C-reactive protein (CRP) was measured using an automated clinical chemistry analyzer (Alinity c, Abbott Laboratories, Chicago, IL, USA), according to the manufacturer’s instructions, expressed in milligrams per liter (mg/L). All analyses were conducted at the accredited clinical laboratories of Debaratana Vejjanukula Hospital Commemorating Her Royal Highness Princess Maha Chakri Sirindhorn’s 60th Birthday Anniversary, Chiang Mai, and Omkoi Hospital, Chiang Mai, under standardized laboratory quality assurance and quality control (QA/QC) procedures.

### 4.3. Leucocyte Telomere Length

Relative leukocyte telomere length (LTL) was determined as the telomere-to-single-copy gene (T/S) ratio using quantitative real-time polymerase chain reaction (qPCR), based on the method described by Cawthon (2009) [56]. Genomic DNA was extracted from peripheral blood leukocytes using the QIAamp DNA Mini Kit (Qiagen, Hilden, Germany). DNA concentration and purity were assessed using a NanoDrop™ 2000 spectrophotometer (Thermo Fisher Scientific, Waltham, MA, USA). For each qPCR reaction, 20 ng of genomic DNA was diluted to a final volume of 20 µL/reaction. Telomere repeats and the single-copy reference gene albumin were quantified using a validated multiplex qPCR assay performed on a QuantStudio™ 5 Real-Time PCR System (Applied Biosystems, Thermo Fisher Scientific). The reaction mixture contained 1× SensiFAST™ SYBR^®^ Lo-ROX Master Mix (2×; Bioline, Meridian Bioscience, Cincinnati, OH, USA) and telomere primer pair TELG/TELC at a final concentration of 900 nM each, together with the albumin primer pair ALBU/ALBD at a final concentration of 900 nM each. Five concentrations of reference DNA (Standard DNA) were prepared using a 4-fold serial dilution from an initial concentration of 100 ng/µL and included on each qPCR plate to generate standard curves for the telomere and single-copy gene targets. Primer sequences for telomeric repeats (TELG and TELC) and the albumin reference gene (ALBU and ALBD) are provided in Supplementary Table S1.

The thermal cycling protocol consisted of three stages: Stage 1, initial denaturation at 95 °C for 15 min; Stage 2, two cycles of 94 °C for 15 s and 49 °C for 15 s; and Stage 3, 35 cycles of 94 °C for 15 s, 62 °C for 10 s, 74 °C for 15 s with fluorescence acquisition, 84 °C for 10 s, and 88 °C for 15 s with fluorescence acquisition. Fluorescence measurements at 74 °C were used to determine the cycle threshold (Ct) values for telomere amplification, whereas measurements at 88 °C were used to determine Ct values for the albumin single-copy gene. At 88 °C, the telomere PCR product was fully melted and therefore did not contribute to the fluorescence signal, allowing specific quantification of the albumin amplification product.

Following amplification, two standard curves were generated for each plate (one for telomere and one for albumin), and the T/S ratio was calculated using the standard-curve method with serial dilutions of genomic DNA obtained from a single individual. The same reference DNA was used across all four analytical batches, with its T/S ratio assigned a value of 1.00 for relative telomere length calculation. Briefly, T represented the amount of standard DNA corresponding to the telomere-repeat copy number of the experimental sample, whereas S represented the amount of standard DNA corresponding to the albumin copy number. The relative telomere length was calculated as the T/S ratio, with the Standard DNA assigned a T/S value of 1.00. Therefore, samples with T/S > 1.00 had relative telomere lengths greater than the Standard DNA, whereas samples with T/S < 1.00 had relative telomere lengths shorter than the Standard DNA. Each experimental DNA sample was analyzed in triplicate, and the mean of the three T/S values was used as the final relative LTL measure in subsequent statistical analyses. The coefficient of variation (CV) of cycle threshold (Ct) values was calculated separately for the telomere and albumin assays to assess variability among technical replicates. Replicate measurements were accepted when the CV was ≤10%; measurements exceeding this threshold were repeated, with a maximum of four attempts in total. Across the four analytical batches, T/S ratios were normalized to the reference DNA sample included in each batch to account for inter-batch variation. To evaluate assay reproducibility, intra-assay and inter-assay coefficients of variation (CVs) were calculated: intra-assay CVs were 0.35% for telomere, 0.33% for albumin, and 1.39% for T/S ratio, while the inter-assay CV for T/S ratio was 9.56%. The 179 participant samples included in the primary analysis were randomly distributed across four analytical batches, with samples from different study sites and blood collection dates represented across batches. Laboratory personnel were separate from those responsible for assessing participants’ PM_2.5_ exposure and did not have access to participants’ exposure data during laboratory analyses. Batch was not included as a covariate in the regression models, and no formal sensitivity analysis was performed to assess potential batch effects. All assays were performed at the Research Center for Molecular and Cell Biology, Research Institute for Health Sciences, Chiang Mai University.

### 4.4. Serum 8-Hydroxy-2′-Deoxyguanosine (8-OHdG) Measurement

Serum 8-hydroxy-2′-deoxyguanosine (8-OHdG) concentrations were determined using a commercially available competitive ELISA kit (Abclonal, Wuhan, China; Cat. No. RK07128) according to the manufacturer’s protocol. Standards, quality-control samples, and serum samples were assayed in duplicate. Absorbance was measured at 450 nm using a CLARIOstar^®^ Plus multimode microplate reader (BMG LABTECH, Ortenberg, Germany), with reference wavelength correction. A standard curve was generated for each assay plate using 8-OHdG standards ranging from 0.78 to 50 ng/mL, and sample concentrations were calculated by interpolation from the corresponding calibration curve and expressed as ng/mL. The assay detection range was 0.78–50 ng/mL, with a reported minimum detectable dose of <0.27 ng/mL. Four assay plates were evaluated for standard-curve performance, with coefficients of determination (R^2^) ranging from 0.961 to 0.991 (mean R^2^ = 0.972). Serum samples were processed according to the manufacturer’s instructions. Samples exceeding the assay range were diluted using the provided sample diluent and reassayed, with the dilution factor incorporated into the final concentration. The intra-assay CV (1.48–6.08%) and inter-batch CV (1.64–9.03%) were both well within the acceptable limit of <10%, demonstrating high precision and reproducibility.

### 4.5. Ambient PM_2.5_ Exposure Assessment

Ambient PM_2.5_ exposure was assessed using daily mean PM_2.5_ concentrations obtained primarily from the Northern Thailand Air Quality and Health Index (NTAQHI) monitoring network. Because PM_2.5_ observations were incomplete for some days or periods at the relevant monitoring sites, GISTDA-derived PM_2.5_ estimates were used to supplement missing observations. The procedures for obtaining, processing, quality controlling, and integrating PM_2.5_ data from the two sources are described below.

Exposure was assigned according to the monitoring site corresponding to each participant’s community and their blood-collection date. Participants resided in communities within an approximate 5-km radius of the corresponding NTAQHI monitoring site; exact participant-specific residence-to-monitor distances were not quantified. Participants sharing the same monitoring site and blood-collection date received identical exposure estimates for each window. Among all 179 participants, these assignments formed eight site–date exposure groups across four monitoring sites—Omkoi, Yang Piang, Mae Na John, and Kong Khak—with 11–32 participants per group.

Cumulative moving-average PM_2.5_ exposures were calculated for five overlapping periods ending on and including the blood-collection date (day 0): 0–7, 0–14, 0–30, 0–60, and 0–90 days. These windows represented 8, 15, 31, 61, and 91 consecutive days, respectively. For each exposure window, the daily PM_2.5_ concentrations were averaged across the corresponding period to obtain the ambient exposure estimate.

#### 4.5.1. NTAQHI Monitoring Network

Ambient PM_2.5_ concentrations were primarily obtained from the Northern Thailand Air Quality and Health Index (NTAQHI) monitoring network operated by the Research Institute for Health Sciences (RIHES), Chiang Mai University. The network uses low-cost optical particle sensors based on PMS7003 sensors (Beijing Plantower Co., Ltd., Beijing, China), which were incorporated into monitoring units developed by Nanogen Company, Chiang Mai, Thailand. The sensors were calibrated and validated against reference-grade air quality monitoring stations, with correction procedures accounting for relative humidity and sensor performance over time. Previous field validation in Chiang Mai demonstrated good agreement between corrected PMS7003 measurements and reference BAM measurements (R^2^ = 0.9737) [44].

The NTAQHI system continuously monitored and recorded PM_2.5_ concentrations, and we retrieved daily mean concentrations from the NTAQHI database for monitoring sites corresponding to the study areas. Data processing, averaging, and quality-control procedures followed standardized RIHES protocols. The NTAQHI measurements served as the primary source of ground-based ambient PM_2.5_ exposure data in this study.

#### 4.5.2. GISTDA-Derived PM_2.5_ Estimates and Data Supplementation

Daily mean PM_2.5_ estimates were obtained from the GISTDA PM_2.5_ monitoring platform (“Check Foon”; https://pm25.gistda.or.th/pm25/, accessed on 14 March 2025), operated by the Geo-Informatics and Space Technology Development Agency, Thailand. The platform integrates satellite information with ground-based monitoring data, and the provider describes a nominal spatial resolution of 1 × 1 km. Estimates were retrieved for selected geographic coordinates within the study subdistricts and matched to NTAQHI observations by calendar date. Before supplementation, GISTDA estimates were adjusted to the local NTAQHI measurement scale using site-specific support vector regression (SVR). Models used daily GISTDA concentrations as the predictor and corresponding NTAQHI measurements as the outcome. The number of paired daily observations was 19 for Omkoi, 43 for Mae Na John, 103 for Kong Khak, and 106 for Yang Piang. Linear and radial basis function (RBF) kernels were evaluated using nested blocked five-fold cross-validation. The kernel yielding the lower cross-validated root mean square error (RMSE) was selected for each site. Linear SVR was selected for Omkoi and Kong Khak. Their fitted equations were estimated NTAQHI PM_2.5_ = 4.6760 + 0.6550 × GISTDA PM_2.5_ and estimated NTAQHI PM_2.5_ = 10.7083 + 0.4854 × GISTDA PM_2.5_, respectively, with concentrations expressed in µg/m^3^. RBF-SVR was selected for Mae Na John and Yang Piang. Predictive performance was assessed using cross-validated RMSE, mean absolute error, mean prediction error, and R^2^. The resulting percentile limits describe uncertainty in the fitted calibration functions rather than full day-level prediction intervals. Predictions outside the range of GISTDA concentrations represented in the paired calibration data were flagged as extrapolations. Predictions from the fitted SVR models were used only to fill missing daily NTAQHI observations; all available NTAQHI measurements were retained. Exposure-window averages and all analyses involving the supplemented exposure dataset were calculated using this calibrated daily dataset. Yang Piang required no supplementation. For each site–date exposure group and exposure window, we recorded the numbers of observed NTAQHI days, GISTDA-supplemented days, and remaining missing days. Supplementation percentages and final data coverage were calculated relative to the intended window lengths of 8, 15, 31, 61, and 91 days, including day 0. These details are reported in Supplementary Table S4.

### 4.6. Statistical Analysis

Statistical analyses were performed using IBM SPSS Statistics 26.0 (IBM Corp., Armonk, NY, USA). SVR calibration and exploratory wild cluster bootstrap analyses were conducted using Python, version 3.11.8. SVR calibration was performed using scikit-learn (version 1.9.1), while the Webb-weighted wild cluster bootstrap procedure was implemented using custom Python code with NumPy (version 2.4.6). The bootstrap procedure used Webb’s six-point weights at the site–date cluster level and CR1-studentized test statistics. Data normality was assessed using the Shapiro–Wilk test. Normally distributed variables were presented as mean ± standard deviation (SD), whereas non-normally distributed variables were presented as median (interquartile range [IQR]). Categorical variables were presented as frequencies and percentages.

Differences between male and female participants were assessed using the Mann–Whitney U test for continuous variables and the chi-square test for categorical variables. For contingency tables with sparse expected cell counts, the Fisher–Freeman–Halton exact test was used, as appropriate.

Associations between ambient PM_2.5_ exposure across multiple exposure windows (lag 0–7, lag 0–14, lag 0–30, lag 0–60, and lag 0–90 days) and clinical or laboratory parameters were evaluated using Spearman’s rank correlation. Spearman’s correlation was also used to examine relationships among leukocyte telomere length, serum 8-hydroxy-2′-deoxyguanosine (8-OHdG), inflammatory biomarkers, and other clinical variables.

No single exposure window was designated as the primary exposure window a priori. Accordingly, comparisons across the five overlapping exposure windows, biomarker analyses, and age-stratified analyses were considered exploratory. For exploratory age-stratified analyses, participants were categorized into three age groups: <35, 35–49, and ≥50 years. The 0–30-day window was used in the hierarchical regression models to examine the incremental contributions of 8-OHdG, SII, and SIRI; this selection should not be interpreted as a prespecified primary exposure window. Associations between estimated ambient PM_2.5_ exposure and relative LTL were examined using covariate-adjusted linear regression. Covariates included age, sex, body mass index (BMI), smoking status, alcohol consumption, biomass-burning exposure, forest-fire exposure, education, occupation, and chronic disease and/or regular medication use. These variables were selected a priori based on biological plausibility, prior epidemiological evidence, and availability in the study dataset [52,57,58].

Regression diagnostics were assessed separately for each of the five exposure-window models. Residual normality was evaluated using the Shapiro–Wilk test, and heteroskedasticity was assessed using the Breusch–Pagan test. Studentized residuals, leverage, and Cook’s distance were used to screen for potentially unusual or influential observations.

To assess the sensitivity of statistical inference to within-group dependence, exploratory wild cluster bootstrap analyses were conducted for the five exposure-window models using Webb weights and 99,999 replications. Clusters were defined by monitoring site and blood-collection date, yielding eight site–date groups among all 179 participants. These models included the same adjustment variables, with categorical covariates represented by indicator variables. Across the full-sample, age-stratified, and sensitivity analyses, coefficients were scaled using the same window-specific exposure IQRs calculated among all 179 participants: 4.63, 6.80, 8.13, 7.25, and 6.25 µg/m^3^ for lag 0–7, 0–14, 0–30, 0–60, and 0–90, respectively. This common scaling facilitated comparison of coefficient magnitudes across analyses. Bootstrap *p*-values were reported separately from conventional OLS *p*-values. For conventional OLS models, unstandardized coefficients (B), standardized coefficients (β), standard errors (SE), and 95% confidence intervals (CI) were reported. Model fit was summarized using R^2^ and adjusted R^2^, with ΔR^2^ reported for hierarchical model comparisons. Conventional tests were two-sided. A *p*-value threshold of 0.05 was used to describe nominal statistical significance. No adjustment for multiple comparisons was applied to any reported *p*-values, including wild cluster bootstrap *p*-values. Accordingly, findings across exposure windows, biomarkers, and age strata were interpreted as exploratory rather than confirmatory.

## 5. Conclusions

PM_2.5_ exposure during the biomass-burning season was associated with higher measured relative LTL for the 0–30-, 0–60-, and 0–90-day windows in conventional covariate-adjusted models. However, estimates varied substantially across sensitivity analyses, and exploratory wild cluster bootstrap analyses using eight site–date groups did not yield statistically significant associations. The findings therefore remain exploratory and do not establish a robust association. Adding serum 8-OHdG and systemic inflammatory indices provided limited additional explanatory information and did not establish mediation by oxidative stress or inflammation. The cross-sectional design, single LTL measurement, shared exposure assignments, and uncertainty in ambient exposure estimation limit interpretation. These findings do not demonstrate within-person telomere change or a causal or protective effect of PM_2.5_. Longitudinal studies incorporating personal exposure assessment, repeated LTL measurements, leukocyte-subset profiling, and more exposure-assignment groups with appropriate methods to address clustering are warranted.

## Figures and Tables

**Table 1:** Characteristics of the study population stratified by sex.

Variable (Median (Q1, Q3))	All (*n* = 179)	Male (*n* = 50)	Female (*n* = 129)	*p*-Value
Age (years)	44.0 (36.0, 51.0)	44.5 (37.8, 51.0)	43.0 (36.0, 51.0)	0.729
Body mass index (BMI) (kg/m^2^)	25.0 (22.8, 28.3)	23.8 (21.5, 26.6)	25.4 (23.7, 28.6)	0.003
Residential duration (years)	37.0 (24.5, 48.0)	34.0 (20.0, 47.0)	37.0 (26.0, 48.0)	0.644
Education level, *n* (%)				0.991
Elementary school or below	62 (34.6)	17 (34.0)	45 (34.9)	
Junior high school	81 (45.3)	23 (46.0)	58 (45.0)	
High school or above	36 (20.1)	10 (20.0)	26 (20.2)	
Occupation (*n*, %)				0.951
Farmer	96 (53.6)	27 (54.0)	69 (53.5)	
Non-farmer	83 (46.4)	23 (46.0)	60 (46.5)	
Smoking (*n*, %)				<0.001
Never smokers	139 (77.7)	14 (28.0)	125 (96.9)	
Former smokers	27 (15.1)	24 (48.0)	3 (2.3)	
Current smokers	13 (7.3)	12 (24.0)	1 (0.8)	
Alcohol consumption (*n*, %)				0.001
Never drinkers	73 (40.8)	11 (22.0)	62 (48.1)	
Current drinkers	106 (59.2)	39 (78.0)	67 (51.9)	
Chronic disease and/or regular medication use				0.847
No chronic disease or regular medication use	127 (70.9)	36 (72.0)	91 (70.5)	
Chronic disease or regular medication use	52 (29.1)	14 (28.0)	38 (29.5)	
–Non-communicable diseases (NCDs)	31 (17.3)	8 (16.0)	23 (17.8)	
–Non-NCD conditions	15 (8.4)	5 (10.0)	10 (7.8)	
–Both NCD and Non-NCD	6 (3.4)	1 (2.0)	5 (3.9)	
Inflammatory biomarkers (median (Q1, Q3))				
C-reactive protein (mg/L)	1.20 (0.50, 2.80)	1.10 (0.58, 2.15)	1.20 (0.50, 2.99)	0.534
Systemic immune-inflammation index (SII) (cells/mm^3^)	459,093 (328,021, 625,291)	430,600 (325,500, 604,216)	479,294 (330,542, 668,158)	0.345
Systemic inflammation response index (SIRI) (cells/mm^3^)	555.0 (387.0, 896.0)	692.0 (450.3, 1058.3)	529.0 (369.5, 806.5)	0.034
Oxidative stress and biological aging biomarkers (median (Q1, Q3))				
Serum 8-hydroxy-2′-deoxyguanosine (ng/mL)	18.0 (15.0, 21.6)	17.3 (13.3, 21.3)	18.0 (15.3, 21.6)	0.318
Leukocyte telomere length	0.81 (0.72, 0.89)	0.81 (0.76, 0.89)	0.81 (0.71, 0.90)	0.691

Note: NCDs included asthma, hypertension, diabetes mellitus, hyperlipidemia, and heart disease. Non-NCD conditions included thyroid disease, peptic ulcer disease, hepatitis B, orthopedic conditions, and allergic conditions. The chronic disease/regular medication category included participants with one or more of these conditions and/or who used regular medication. Participants could have conditions from both NCD and non-NCD categories.

**Table 2:** Spearman correlations of PM_2.5_ exposure with Inflammatory biomarkers, oxidative stress, and Leukocyte telomere length.

Variable		Lag 0–7	Lag 0–14	Lag 0–30	Lag 0–60	Lag 0–90
PM_2.5_ (µg/m^3^)	Median Q1, Q3 IQR Min–Max	58.4 (55.4–60.0)	49.3 (44.4–51.2)	44.0 (42.7–50.9)	34.6 (33.6–40.9)	31.0 (27.7–34.0)
		4.6 53.8–66.7	6.8 42.8–65.8	8.1 41.7–57.5	7.3 32.7–43.8	6.3 27.2–36.1
C-reactive protein		−0.096 (0.201)	−0.043 (0.570)	−0.005 (0.947)	0.015 (0.840)	0.040 (0.598)
Systemic immune-inflammation index		0.016 (0.829)	0.005 (0.947)	−0.110 (0.142)	−0.174 (0.020)	−0.191 (0.011)
Systemic inflammation response index		−0.092 (0.220)	−0.070 (0.349)	−0.189 (0.011)	−0.220 (0.003)	−0.204 (0.006)
Serum 8-OHdG		−0.108 (0.148)	−0.065 (0.389)	0.123 (0.100)	0.127 (0.089)	0.129 (0.086)
Leukocyte telomere length		0.027 (0.718)	−0.030 (0.692)	0.227 (0.002)	0.253 (0.001)	0.243 (0.001)

**Table 3:** Multivariable association of PM_2.5_ exposure and leukocyte telomere length.

PM_2.5_ Exposure	Adjusted (per 10 µg/m^3^)			Adjusted (per IQR)			Standardized β	*p*-Value
	B	Standard Error (SE)	95% CI	B	Standard Error (SE)	95% CI		
Lag 0–7	−0.014	0.035	−0.083 to 0.055	−0.006	0.016	−0.03 to 0.026	−0.033	0.690
Lag 0–14	−0.015	0.019	−0.052 to 0.021	−0.010	0.013	−0.035 to 0.014	−0.071	0.407
Lag 0–30	0.062	0.024	0.014 to 0.110	0.050	0.020	0.012 to 0.089	0.218	0.011
Lag 0–60	0.108	0.031	0.048 to 0.169	0.079	0.022	0.035 to 0.123	0.289	<0.001
Lag 0–90	0.151	0.043	0.066 to 0.233	0.094	0.027	0.041 to 0.147	0.298	<0.001

**Table 4:** Exploratory hierarchical linear regression models of 0–30-day PM_2.5_ exposure, oxidative stress, inflammatory indices, and relative leukocyte telomere length (*n* = 179).

Model and Step	Model 1:	Model 2:		Model 3A:			Model 3B:			Model 3C:		
	Covariates	Covariates + 0–30-Day PM_2.5_		Covariates			Covariates			Covariates		
				+ 0–30-Day PM_2.5_			+ 0–30-Day PM_2.5_			+ 0–30-Day PM_2.5_		
				+ 8-OHdG			+ SII			+ SIRI		
Predictor	-	PM_2.5_,		PM_2.5_,		8-OHdG,	PM_2.5_,		SII,	PM_2.5_,		SIRI,
		per 10 µg/m^3^	per IQR	per 10 µg/m^3^	per IQR	per ng/mL	per 10 µg/m^3^	per IQR	per cells/mm^3^	per 10 µg/m^3^	per IQR	per cells/mm^3^
B	-	0.062	0.050	0.058	0.047	0.004	0.061	0.049	−3.221 × 10^−8^	0.062	0.050	−1.366 × 10^−5^
(95% CI)		(0.014–0.110)	(0.012 to 0.089)	(0.010–0.105)	(0.008 to 0.085)	(0.000–0.008)	(0.013–0.109)	(0.011 to 0.088)	(−1.261 × 10^−7^ to 6.173 × 10^−8^)	(0.014 to 0.109)	(0.011 to 0.089)	(−6.547 × 10^−5^ to 3.815 × 10^−5^)
Standardized β	-	0.218		0.203		0.155	0.214		−0.053	0.216		−0.041
*p*	-	0.011		0.017		0.041	0.013		0.499	0.012		0.603
R^2^ (adjusted R^2^)	0.018 (−0.041)	0.055 (−0.007)		0.079 (0.012)		-	0.058 (−0.010)		-	0.057 (−0.012)		-
ΔR^2^; F-change (*p*)	0.018; 0.301 (0.980)	0.038; 6.637 (0.011)		0.024; 4.246 (0.041)		-	0.003; 0.458 (0.499)		-	0.002; 0.271 (0.603)		-
Overall model *p*	0.980	0.555		0.300		-	0.601		-	0.618		-

Models 3A–3C represent separate alternative extensions of Model 2 and were not entered sequentially. Model 3A added serum 8-OHdG, Model 3B added SII, and Model 3C added SIRI to the variables included in Model 2. The ΔR^2^ and F-change-test *p*-values for Models 3A–3C were calculated relative to Model 2. For Model 1, ΔR^2^ represents the change from the intercept-only model.

## Data Availability

The original data were generated for this study but are not publicly available due to privacy and ethical restrictions. The data may be made available from the corresponding author upon reasonable request, subject to the applicable restrictions.

## Supplementary Materials

The following supporting information can be downloaded at https://www.mdpi.com/article/10.3390/ijms27219869/s1.

## Abbreviations

The following abbreviations are used in this manuscript:

8-OHdG	8-Hydroxy-2′-deoxyguanosine
BMI	Body mass index
CBC	Complete blood count
CI	Confidence interval
CRP	C-reactive protein
IQR	Interquartile range
LTL	Leukocyte telomere length
NTAQHI	Northern Thailand Air Quality and Health Index
PAHs	Polycyclic aromatic hydrocarbons
PM_2.5_	Particulate matter with an aerodynamic diameter of ≤2.5 μm
qPCR	Quantitative polymerase chain reaction
QA/QC	Quality assurance and quality control
SD	Standard deviation
SE	Standard error
SII	Systemic immune-inflammation index
SIRI	Systemic inflammation response index
T/S ratio	Telomere-to-single-copy gene ratio

## References

[1] Brauer M., Roth G.A., Aravkin A.Y., Zheng P., Abate K.H., Abate Y.H., Abbafati C., Abbasgholizadeh R., Abbasi M.A., Abbasian M. (2024). Global burden and strength of evidence for 88 risk factors in 204 countries and 811 subnational locations, 1990–2021: A systematic analysis for the Global Burden of Disease Study 2021. Lancet.

[2] Schraufnagel D.E., Balmes J.R., Cowl C.T., De Matteis S., Jung S.H., Mortimer K., Perez-Padilla R., Rice M.B., Riojas-Rodriguez H., Sood A. (2019). Air Pollution and Noncommunicable Diseases: A Review by the Forum of International Respiratory Societies’ Environmental Committee, Part 1: The Damaging Effects of Air Pollution. Chest.

[3] World Health Organization (2021). WHO Global Air Quality Guidelines: Particulate Matter (PM2.5 and PM10), Ozone, Nitrogen Dioxide, Sulfur Dioxide and Carbon Monoxide.

[4] Li Y., Sun P., Yin Y., Yu C., Xie D., Wan Z., Deng B. (2026). Global, Regional, and National Burden Attributed to Particulate Matter Pollution, 1990–2021: A Systematic Analysis for the Global Burden of Disease Study 2021. Ann. Glob. Health.

[5] Wang S.N., Shi Y.C., Lin S., He H.F. (2024). Particulate matter 2.5 accelerates aging: Exploring cellular senescence and age-related diseases. Ecotoxicol. Environ. Saf..

[6] Yin S., Wang X., Zhang X., Guo M., Miura M., Xiao Y. (2019). Influence of biomass burning on local air pollution in mainland Southeast Asia from 2001 to 2016. Environ. Pollut..

[7] Chansuebsri S., Kraisitnitikul P., Wiriya W., Chantara S. (2022). Fresh and aged PM(2.5) and their ion composition in rural and urban atmospheres of Northern Thailand in relation to source identification. Chemosphere.

[8] Chansuebsri S., Kolar P., Kraisitnitikul P., Kantarawilawan N., Yabueng N., Wiriya W., Thepnuan D., Chantara S. (2024). Chemical composition and origins of PM2.5 in Chiang Mai (Thailand) by integrated source apportionment and potential source areas. Atmos. Environ..

[9] Thongsame W., Henze D.K., Barth M., Pfister G., Kumar R., Macatangay R., Hassan Bran S. (2025). Source Attribution and Health Burden of PM2.5 in Mainland Thailand. GeoHealth.

[10] Suriyawong P., Chuetor S., Samae H., Piriyakarnsakul S., Amin M., Furuuchi M., Hata M., Inerb M., Phairuang W. (2023). Airborne particulate matter from biomass burning in Thailand: Recent issues, challenges, and options. Heliyon.

[11] Chotamonsak C., Thanadolmethaphorn P., Lapyai D., Chimla S. (2026). Near Real-Time Biomass Burning PM2.5 Emission Estimation to Support Environmental Health Risk Management in Northern Thailand Using FINNv2.5. Toxics.

[12] Yabueng N., Wiriya W., Chantara S. (2020). Influence of zero-burning policy and climate phenomena on ambient PM2.5 patterns and PAHs inhalation cancer risk during episodes of smoke haze in Northern Thailand. Atmos. Environ..

[13] Ruiz A., Flores-Gonzalez J., Buendia-Roldan I., Chavez-Galan L. (2022). Telomere Shortening and Its Association with Cell Dysfunction in Lung Diseases. Int. J. Mol. Sci..

[14] Yeh J.-K., Wang C.-Y. (2016). Telomeres and Telomerase in Cardiovascular Diseases. Genes.

[15] Gruber H.-J., Semeraro M.D., Renner W., Herrmann M. (2021). Telomeres and Age-Related Diseases. Biomedicines.

[16] Patel C.J., Manrai A.K., Corona E., Kohane I.S. (2017). Systematic correlation of environmental exposure and physiological and self-reported behaviour factors with leukocyte telomere length. Int. J. Epidemiol..

[17] Martens D.S., Nawrot T.S. (2018). Ageing at the level of telomeres in association to residential landscape and air pollution at home and work: A review of the current evidence. Toxicol. Lett..

[18] Baylie T., Gugsa E., Jemal M., Baye G., Getinet M., Amare G.A., Adugna A., Abebaw D., Hibstu Z., Tegegne B.A. (2026). Telomerase and chronic inflammation as central molecular links in aging. Biomed. Pharmacother..

[19] Miri M., Nazarzadeh M., Alahabadi A., Ehrampoush M.H., Rad A., Lotfi M.H., Sheikhha M.H., Sakhvidi M.J.Z., Nawrot T.S., Dadvand P. (2019). Air pollution and telomere length in adults: A systematic review and meta-analysis of observational studies. Environ. Pollut..

[20] Wei B., Zhou Y., Li Q., Zhen S., Wu Q., Xiao Z., Liao J., Zhu B., Duan J., Yang X. (2024). Outdoor fine particulate matter exposure and telomere length in humans: A systematic review and meta-analysis. Ecotoxicol. Environ. Saf..

[21] Hautekiet P., Nawrot T.S., Martens D.S., Bijnens E.M., De Keersmaecker S.C., Van der Heyden J., De Clercq E.M., Saenen N.D. (2025). Recent and chronic ambient air pollution exposure in association with telomere length and mitochondrial DNA content in the general population. Environ. Res..

[22] Hou L., Wang S., Dou C., Zhang X., Yu Y., Zheng Y., Avula U., Hoxha M., Díaz A., McCracken J. (2012). Air pollution exposure and telomere length in highly exposed subjects in Beijing, China: A repeated-measure study. Environ. Int..

[23] Chang-Chien J., Kuo M.L., Tseng Y.L., Huang H.Y., Tsai H.J., Yao T.C. (2024). Differential effects of long- and short-term exposure to PM2.5 on accelerating telomere shortening: From in vitro to epidemiological studies. Ecotoxicol. Environ. Saf..

[24] Chen B.H., Carty C.L., Kimura M., Kark J.D., Chen W., Li S., Zhang T., Kooperberg C., Levy D., Assimes T. (2017). Leukocyte telomere length, T cell composition and DNA methylation age. Aging.

[25] Carugno M., Borroni E., Fedrizzi L., Hoxha M., Vigna L., Consonni D., Bollati V., Pesatori A.C. (2021). Long- and Short-Term Exposures to PM10 Can Shorten Telomere Length in Individuals Affected by Overweight and Obesity. Life.

[26] Dioni L., Hoxha M., Nordio F., Bonzini M., Tarantini L., Albetti B., Savarese A., Schwartz J., Bertazzi P.A., Apostoli P. (2011). Effects of short-term exposure to inhalable particulate matter on telomere length, telomerase expression, and telomerase methylation in steel workers. Environ. Health Perspect..

[27] Xu F., Shi X., Qiu X., Jiang X., Fang Y., Wang J., Hu D., Zhu T. (2020). Investigation of the chemical components of ambient fine particulate matter (PM2.5) associated with in vitro cellular responses to oxidative stress and inflammation. Environ. Int..

[28] Barnes R.P., Fouquerel E., Opresko L. (2019). The impact of oxidative DNA damage and stress on telomere homeostasis. Mech. Ageing Dev..

[29] Wong J.Y., De Vivo I., Lin X., Fang S.C., Christiani D.C. (2014). The relationship between inflammatory biomarkers and telomere length in an occupational prospective cohort study. PLoS ONE.

[30] Di Minno A., Turnu L., Porro B., Squellerio I., Cavalca V., Tremoli E., Di Minno M.N.D. (2016). 8-Hydroxy-2-Deoxyguanosine Levels and Cardiovascular Disease: A Systematic Review and Meta-Analysis of the Literature. Antioxid. Redox Signal..

[31] Cho C.-C., Hsieh W.Y., Tsai C.H., Chen C.Y., Chang H.F., Lin C.S. (2018). In Vitro and In Vivo Experimental Studies of PM2.5 on Disease Progression. Int. J. Environ. Res. Public Health.

[32] Ke J., Wu K., Liu X., Qiu H., Liu Q., Song J., Liu Y., Ruan X., Zhou Y. (2026). Impact of systemic immune-inflammation index and systemic inflammation response index on all-cause and cause-specific mortality: A community-based cohort study. Front. Med..

[33] Kim J.H., Woo H.D., Lee J.J., Song D.S., Lee K. (2024). Association between short-term exposure to ambient air pollutants and biomarkers indicative of inflammation and oxidative stress: A cross-sectional study using KoGES-HEXA data. Environ. Health Prev. Med..

[34] Pilger A., Rüdiger H.W. (2006). 8-Hydroxy-2′-deoxyguanosine as a marker of oxidative DNA damage related to occupational and environmental exposures. Int. Arch. Occup. Environ. Health.

[35] Ren C., Fang S., Wright R.O., Suh H., Schwartz J. (2011). Urinary 8-hydroxy-2′-deoxyguanosine as a biomarker of oxidative DNA damage induced by ambient pollution in the Normative Aging Study. Occup. Environ. Med..

[36] Xia Y., Xia C., Wu L., Li Z., Li H., Zhang J. (2023). Systemic Immune Inflammation Index (SII), System Inflammation Response Index (SIRI) and Risk of All-Cause Mortality and Cardiovascular Mortality: A 20-Year Follow-Up Cohort Study of 42,875 US Adults. J. Clin. Med..

[37] Yang C.-H., Wang X.Y., Zhang Y.H., Ding N. (2025). SIRI and SII as potential biomarkers of disease activity and lupus nephritis in systemic lupus erythematosus. Front. Immunol..

[38] National Environment Board (2026). Notification of the National Environment Board on Ambient Air Quality Standards, B.E. 2569 (2026).

[39] Jarernwong K., Gheewala S.H., Sampattagul S. (2023). Health Impact Related to Ambient Particulate Matter Exposure as a Spatial Health Risk Map Case Study in Chiang Mai, Thailand. Atmosphere.

[40] Mueller W., Loh M., Vardoulakis S., Johnston H.J., Steinle S., Precha N., Kliengchuay W., Tantrakarnapa K., Cherrie J.W. (2020). Ambient particulate matter and biomass burning: An ecological time series study of respiratory and cardiovascular hospital visits in northern Thailand. Environ. Health.

[41] Ngamsang P., Amnuaylojaroen T., Parasin N., Pimonsree S. (2023). Health Impact Assessment of Short-Term Exposure to Particulate Matter (PM10) in Northern Thailand. J. Environ. Public Health.

[42] Dejchanchaiwong R., Tekasakul P., Saejio A., Limna T., Le T.C., Tsai C.J., Lin G.Y., Morris J. (2023). Seasonal Field Calibration of Low-Cost PM2.5 Sensors in Different Locations with Different Sources in Thailand. Atmosphere.

[43] Yu T., Wang W., Ciren P., Sun R. (2018). An assessment of air-quality monitoring station locations based on satellite observations. Int. J. Remote Sens..

[44] Kumpika T., Sroila W., Intra P., Kawichai S., Prapamontol T., Thongsuwan W., Singjai P. (2026). Correction of humidity and sensor aging effects in low-cost PM2.5 light scattering sensors for improved measurement accuracy. Environ. Pollut. Bioavailab..

[45] Autsavapromporn N., Klunklin P., Threeratana C., Tuntiwechapikul W., Hosoda M., Tokonami S. (2018). Short Telomere Length as a Biomarker Risk of Lung Cancer Development Induced by High Radon Levels: A Pilot Study. Int. J. Environ. Res. Public Health.

[46] Winson P., Leelahagul P., Taweewongsounton A., Puenputtho W., Bovornkitti S., Poungvarin N. (2022). Telomere length in Thai Buddhist monks and Thai males aged 40 years and above. Malays. J. Nutr..

[47] Nantanawut T., Leelahagul P., Taweewongsounton A., Puengputtho W., Bovornkitti S. (2022). Telomere Length in Various Age Groups of Normal-Body Weight Thais and Obese Thais. Asian Med. J. Altern. Med..

[48] Lin J., Smith D.L., Esteves K., Drury S. (2019). Telomere length measurement by qPCR—Summary of critical factors and recommendations for assay design. Psychoneuroendocrinology.

[49] Morinha F., Magalhães P., Blanco G. (2020). Standard guidelines for the publication of telomere qPCR results in evolutionary ecology. Mol. Ecol. Resour..

[50] Pieters N., Janssen B.G., Dewitte H., Cox B., Cuypers A., Lefebvre W., Smeets K., Vanpoucke C., Plusquin M., Nawrot T.S. (2016). Biomolecular Markers within the Core Axis of Aging and Particulate Air Pollution Exposure in the Elderly: A Cross-Sectional Study. Environ. Health Perspect..

[51] Li S., Yang M., Carter E., Schauer J.J., Yang X., Ezzati M., Goldberg M.S., Baumgartner J. (2019). Exposure–Response Associations of Household Air Pollution and Buccal Cell Telomere Length in Women Using Biomass Stoves. Environ. Health Perspect..

[52] Zhao B., Vo H.Q., Johnston F.H., Negishi K. (2018). Air pollution and telomere length: A systematic review of 12,058 subjects. Cardiovasc. Diagn. Ther..

[53] Wu Y., Gasevic D., Wen B., Yu P., Xu R., Zhou G., Zhang Y., Song J., Liu H., Li S. (2023). Association between air pollution and telomere length: A study of 471,808 UK Biobank participants. Innov. Med..

[54] Commodore A.A., Zhang J.J., Chang Y., Hartinger S.M., Lanata C.F., Mäusezahl D., Gil A.I., Hall D.B., Aguilar-Villalobos M., Vena J.E. (2013). Concentrations of urinary 8-hydroxy-2′-deoxyguanosine and 8-isoprostane in women exposed to woodsmoke in a cookstove intervention study in San Marcos, Peru. Environ. Int..

[55] Islam M.M., Satici M.O., Eroglu S.E. (2024). Unraveling the clinical significance and prognostic value of the neutrophil-to-lymphocyte ratio, platelet-to-lymphocyte ratio, systemic immune-inflammation index, systemic inflammation response index, and delta neutrophil index: An extensive literature review. Turk. J. Emerg. Med..

[56] Cawthon R.M. (2009). Telomere length measurement by a novel monochrome multiplex quantitative PCR method. Nucleic Acids Res..

[57] Müezzinler A., Zaineddin A.K., Brenner H. (2014). Body mass index and leukocyte telomere length in adults: A systematic review and meta-analysis. Obes. Rev..

[58] Astuti Y., Wardhana A., Watkins J., Wulaningsih W. (2017). Cigarette smoking and telomere length: A systematic review of 84 studies and meta-analysis. Environ. Res..

