# Supplementary material for: Association Between Ambient PM2.5 Exposure and Leukocyte Telomere Length Among Rural Adults: Cross-Sectional Study in Northern Thailand
Source: Int J Mol Sci. 2026 Nov 4;27(21):9869. doi: 10.3390/ijms27219869 (PMC13764041; doi:10.3390/ijms27219869)
Supplement: Supplementary file 1 [file ijms-27-09869-s001.zip › ijms-4544234-supplementary.pdf]

Association between Ambient $PM_{2.5}$ Exposure and Leukocyte Telomere Length Among Rural Adults: Cross-Sectional Study in Northern Thailand

Kanyapak Kohsuwan[1,2], Sayamon Hongjaisee[1,2], Kawinwut Somsunun[1,3], Nattawadee Promkam[1,2], Teetawat Santijitpakdee[1,2] and Tippawan Prapamontol[1*]

Supplementary

Figure S1. Participant selection and analytical flow. Of the 194 individuals assessed for eligibility, 15 were excluded because they had incomplete study data or biological sample collection (n = 11) and unstable non-communicable diseases (n = 4). The final analysis included 179 participants with complete exposure, covariate, and leukocyte telomere-length data. Participants comprised eight site–date groups across four areas: Omkoi (n = 27; 11 and 16), Yang Piang (n = 49; 25 and 24), Mae Na John (n = 61; 32 and 29), and Kong Khak (n = 42; 24 and 18).

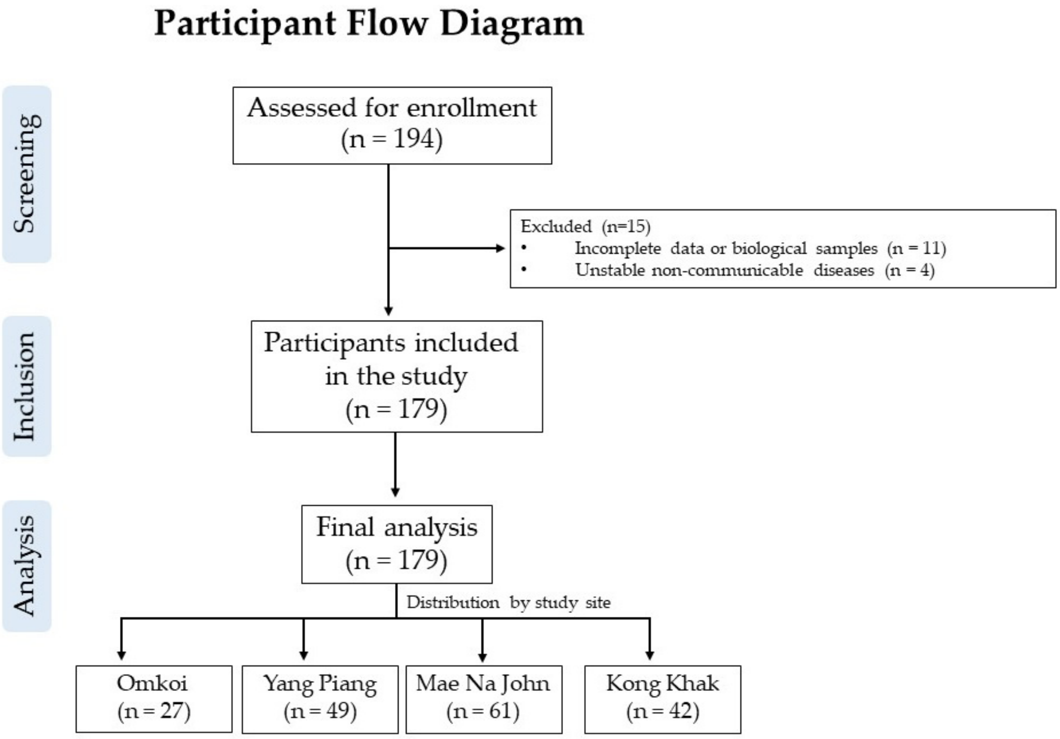

Table S1. Primer sequences used for quantitative PCR analysis of relative telomere length#

Oligo Name	Target	5'- Oligo Seq -3'
TELG	telomeric repeats	ACACTAAGGTTTGGGTTTGGGTTTG GGTTTGGGTTAGTGT
TELC	telomeric repeats	TGTTAGGTATCC CTATCCCTATCCCTATCCCTATCCCTAACA
ALBU	reference gene	CGGCGGCGGGCGGCGCGGGCTGGGCGGAAATGCTGCACAGAATCCTTG
ALBD	reference gene	GCCCGGCCCGCCGCG CCCGTCCCGCCGGAAAAGCATGGTCGCCTGTT

Table S2. qPCR assay performance and reproducibility

Assay parameter	Telomere	Albumin	T/S ratio
Analytical batches (n)	4	4	4
Intra-assay CV (%)	0.35	0.33	1.39
Inter-assay CV (%)	—	—	9.56
Reaction efficiency (%)	106.13	100.7	—

Table S3. Number of available daily $PM_{2.5}$ observations before and after GISTDA supplementation.

Site	NTAQHI available (n)	Missing NTAQHI (n)	Missing NTAQHI (%)	GISTDA supplemented (n)	Final (n)
Omkoi	19	87	82.1	87	106
Mae Na John	43	63	59.4	63	106
Yang Piang	106	0	0.0	0	106
Kong Khak	103	3	2.8	3	106

Table S4. Completeness of $PM_{2.5}$ Exposure Data and Percentage of GISTDA-Supplemented Days by Site–Date Group.

Site–date group	0–7 days	0–14 days	0–30 days	0–60 days	0–90 days
Omkoi / Day 1	7/8 (87.5%)	14/15 (93.3%)	30/31 (96.8%)	60/61 (98.4%)	87/91 (95.6%)
Omkoi / Day 2	6/8 (75.0%)	13/15 (86.7%)	29/31 (93.5%)	59/61 (96.7%)	87/91 (95.6%)
Mae Na John / Day 1	0/8 (0.0%)	0/15 (0.0%)	1/31 (3.2%)	31/61 (50.8%)	61/91 (67.0%)
Mae Na John / Day 2	0/8 (0.0%)	0/15 (0.0%)	0/31 (0.0%)	30/61 (49.2%)	60/91 (65.9%)
Yang Piang / Day 1	0/8 (0.0%)	0/15 (0.0%)	0/31 (0.0%)	0/61 (0.0%)	0/91 (0.0%)
Yang Piang / Day 2	0/8 (0.0%)	0/15 (0.0%)	0/31 (0.0%)	0/61 (0.0%)	0/91 (0.0%)
Kong Khak / Day 1	0/8 (0.0%)	0/15 (0.0%)	0/31 (0.0%)	3/61 (4.9%)	3/91 (3.3%)
Kong Khak / Day 2	0/8 (0.0%)	0/15 (0.0%)	0/31 (0.0%)	3/61 (4.9%)	3/91 (3.3%)

Table S5. Descriptive statistics and linear regression comparison of $PM_{2.5}$ concentrations before and after supplementation with GISTDA-derived data.

Site	Dataset	n	Mean ± SD (μg/m³)	Min–Max (μg/m³)	Median (μg/m³)	Q1	Q3	CV (%)	Slope (β)	Intercept (α)	R^2	RMSE (μg/m³)	P value	95% CI for slope
Omkoi	NTAQHI	19	69.00 ± 27.95	25.00–127.00	68.0	42.0	90.0	40.5	1.017	25.120	0.714	17.509	<0.0001	0.6880–1.347
	NTAQHI + GISTDA	106	39.31 ± 26.09	7.50–127.00	32.1	18.7	51.8	66.4	1.311	2.891	0.944	8.319	<0.0001	1.249–1.373
Yang Piang	NTAQHI	106	31.22 ± 19.58	5.00–93.00	24.0	18.0	39.5	62.7	1.454	10.170	0.796	14.363	<0.0001	1.311–1.598
	NTAQHI + GISTDA	106	31.22 ± 19.58	5.00–93.00	24.0	18.0	39.5	62.7	1.454	10.170	0.796	14.363	<0.0001	1.311–1.598
Mae Na John	NTAQHI	43	49.65 ± 28.04	15.00–152.00	41.0	28.0	70.0	56.5	1.135	35.340	0.739	18.709	<0.0001	0.9225–1.348
	NTAQHI + GISTDA	106	29.48 ± 25.33	5.23–152.00	22.1	11.2	36.9	85.9	1.505	9.630	0.865	15.016	<0.0001	1.389–1.620
Kong Khak	NTAQHI	103	35.43 ± 19.73	11.00–98.00	29.0	21.0	48.0	55.7	1.719	-11.580	0.860	13.626	<0.0001	1.582–1.856
	NTAQHI + GISTDA	106	35.21 ± 19.49	11.00–98.00	28.5	21.0	47.2	55.4	1.720	-11.680	0.861	13.441	<0.0001	1.585–1.855

Abbreviations: NTAQHI, Northern Thailand Air Quality and Health Index; GISTDA, Geo-Informatics and Space Technology Development Agency; RMSE, root mean square error; R^2, coefficient of determination.

Figure S2. Linear relationships between NTAQHI-measured and GISTDA-derived daily $PM_{2.5}$ concentrations across the study sites.

NTAQHI observations only | NTAQHI + GISTDA/supplemented observations

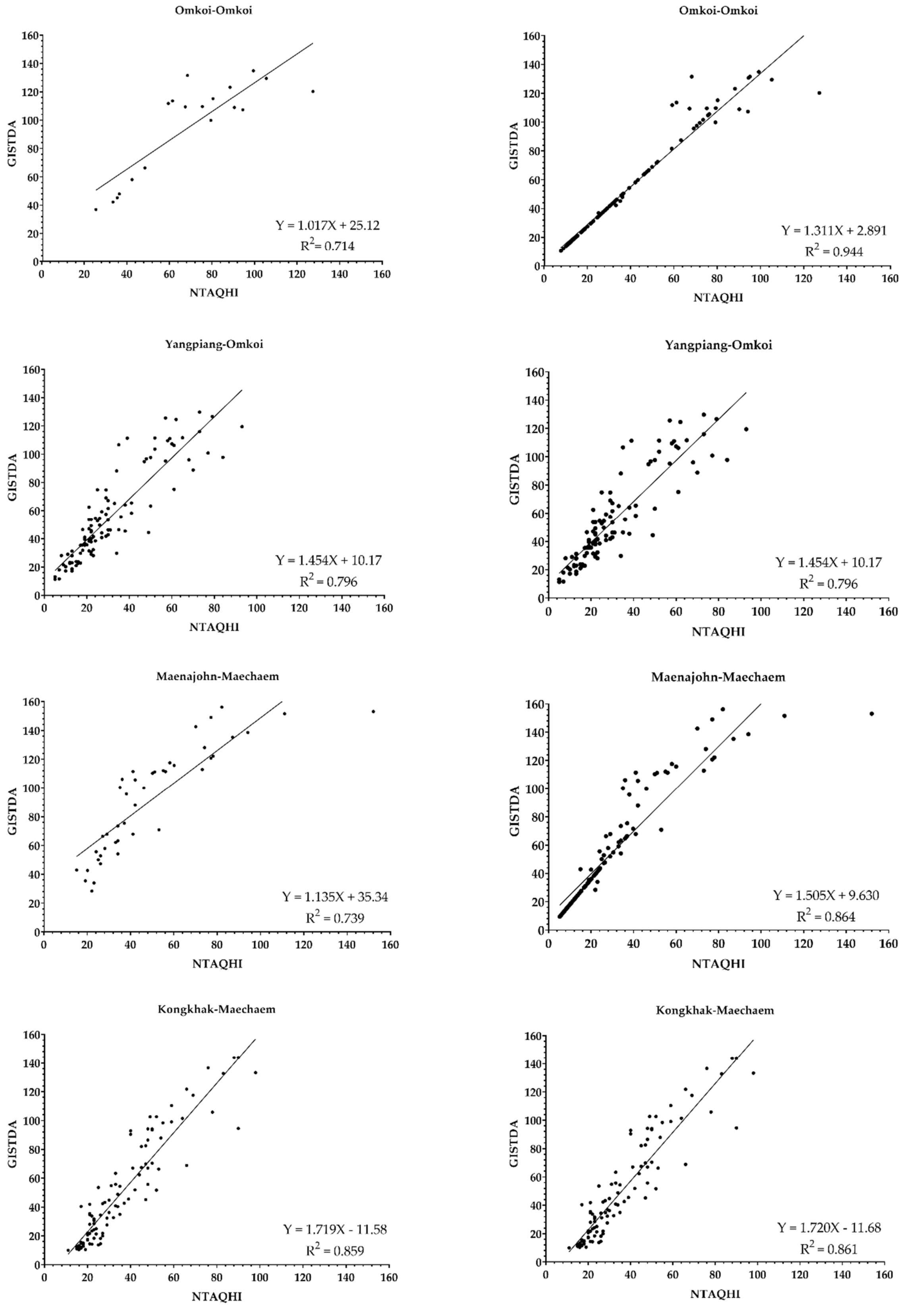

Figure S3. Daily $PM_{2.5}$ concentrations before and after data supplementation using GISTDA-derived estimates.

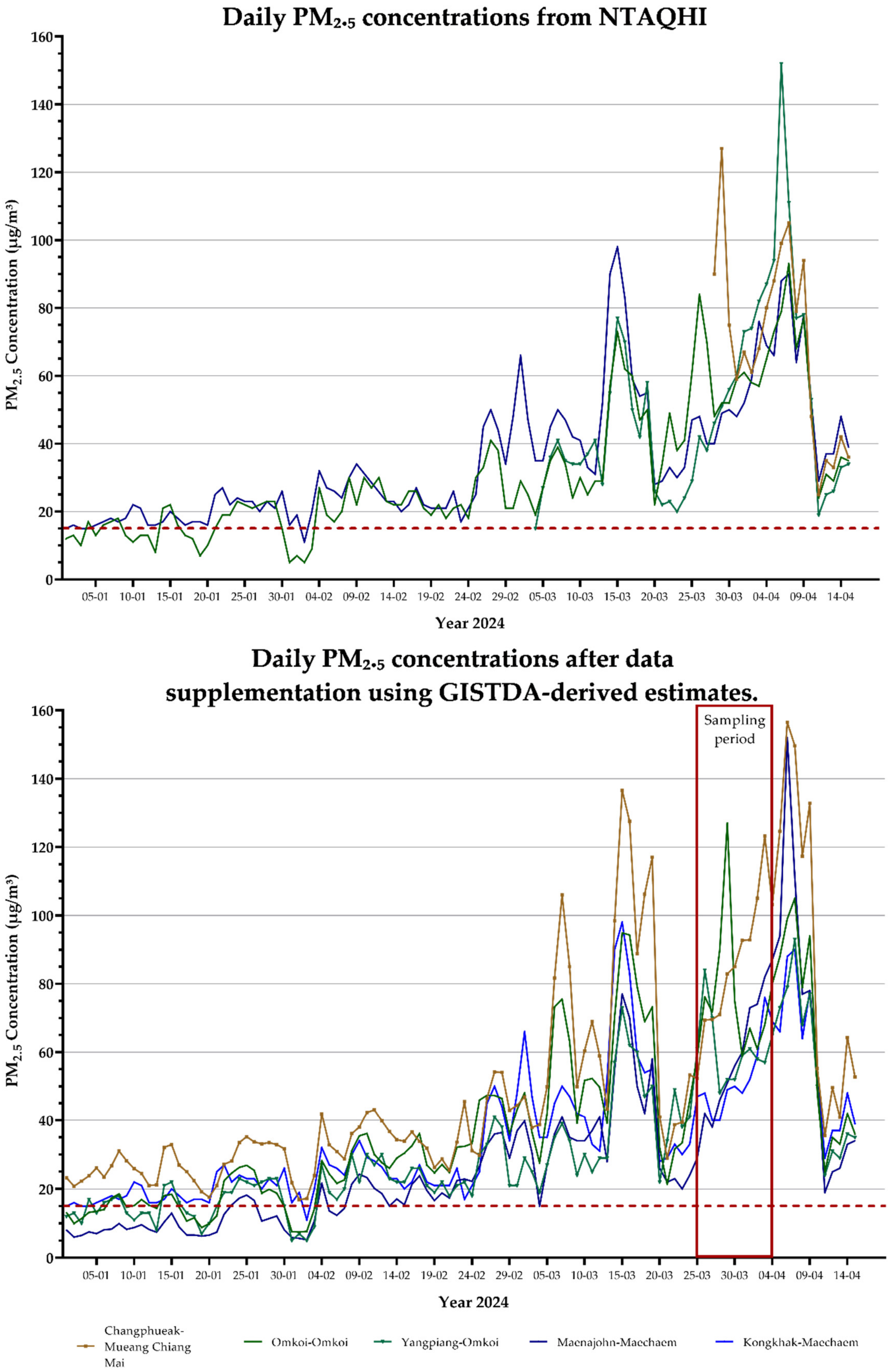

Table S6. Age-stratified associations between $PM_{2.5}$ exposure and relative leukocyte telomere length across cumulative exposure windows

$PM_{2.5}$ exposure	<35 years B (SE)			35–49 years B (SE)			≥ 50 years B (SE)		
	**per 10 μg/m³**	**per IQR**	**(p-value)**	**per 10 μg/m³**	**per IQR**	**(p-value)**	**per 10 μg/m³**	**per IQR**	**(p-value)**
Lag 0–7	-0.051 (0.187)	-0.0236 (0.0865)	0.787	0.028 (0.062)	0.0130 (0.0287)	0.657	-0.066 (0.046)	-0.0305 (0.0213)	0.158
Lag 0–14	0.127 (0.111)	0.0864 (0.0755)	0.266	-0.053 (0.032)	-0.0360 (0.0218)	0.097	-0.021 (0.027)	-0.0143 (0.0184)	0.451
Lag 0–30	0.333 (0.054)	0.2707 (0.0439)	<0.001	0.048 (0.041)	0.0390 (0.0333)	0.253	0.006 (0.041)	0.0049 (0.0333)	0.891
Lag 0–60	0.353 (0.060)	0.2559 (0.0435)	<0.001	0.095 (0.051)	0.0689 (0.0370)	0.065	0.033 (0.060)	0.0239 (0.0435)	0.579
Lag 0–90	0.391 (0.089)	0.2445 (0.0556)	<0.001	0.134 (0.068)	0.0838 (0.0425)	0.051	0.040 (0.095)	0.0250 (0.0594)	0.675

Note: B is the unstandardized regression coefficient, expressed as the difference in relative LTL per 10 μg/m³ or per IQR increase in PM2.5 exposure, as indicated in the column headings; SE, standard error. Models were adjusted for sex, age, BMI, smoking, alcohol consumption, biomass-burning exposure, forest-fire exposure, education, occupation, and chronic disease/regular medication use. All p-values are unadjusted for multiple comparisons.

Table S7. Sensitivity analyses of the association between PM2.5 exposure and relative leukocyte telomere length

$PM_{2.5}$ exposure	Primary (n= 179)				Exclude Omkoi (n=152)				NTAQHI-only (n=91)			
	$PM_{2.5,}$ μg/m³ Median (IQR)	B (95% CI) per 10 μg/m³	B (95% CI) per IQR	p-value	$PM_{2.5,}$ μg/m³ Median (IQR)	B (95% CI) per 10 μg/m³	B (95% CI) per IQR	p-value	$PM_{2.5,}$ μg/m³ Median (IQR)	B (95% CI) per 10 μg/m³	B (95% CI) per IQR	p-value
Lag 0–7	58.38 (4.63)	−0.014 (−0.083 to 0.055)	−0.006 (−0.038 to 0.025)	0.690	58.38 (4.63)	−0.009 (−0.125 to 0.107)	−0.004 (−0.058 to 0.049)	0.879	58.38 (5.50)	−0.213 (−0.409 to −0.017)	−0.099 (−0.189 to −0.008)	0.036
Lag 0–14	49.33 (6.80)	−0.015 (−0.052 to 0.021)	−0.010 (−0.035 to 0.014)	0.407	46.87 (6.73)	0.011 (−0.069 to 0.091)	0.007 (−0.047 to 0.062)	0.79	51.13 (4.33)	−0.416 (−0.614 to −0.218)	−0.283 (−0.418 to −0.148)	<0.001
Lag 0–30	44.03 (8.13)	0.062 (0.015 to 0.109)	0.050 (0.012 to 0.089)	0.011	43.42 (7.13)	0.179 (0.114 to 0.244)	0.146 (0.093 to 0.198)	<0.001	44.03 (7.13)	0.249 (0.165 to 0.333)	0.202 (0.134 to 0.271)	<0.001
Lag 0–60	34.57 (7.25)	0.108 (0.048 to 0.169)	0.078 (0.035 to 0.123)	<0.001	33.64 (6.69)	0.202	0.146 (0.095 to 0.198)	<0.001	33.64 (7.59)	0.234 (0.154 to 0.314)	0.170 (0.112 to 0.228)	<0.001
Lag 0–90	30.97 (6.25)	0.151 (0.067 to 0.235)	0.094 (0.042 to 0.147)	<0.001	30.32 (5.70)	0.254	0.159 (0.100 to 0.218)	<0.001	27.71 (6.24)	0.277 (0.181 to 0.373)	0.173 (0.113 to 0.233)	<0.001

Note: B is the unstandardized regression coefficient, expressed as the difference in relative LTL. Coefficients are reported per 10 μg/m³ and per IQR increase in $PM_{2.5}$ exposure. Models retained the original covariate adjustment. Confidence intervals and p-values are based on conventional regression inference and are unadjusted for site–date clustering and multiple comparisons. The primary analysis used the supplemented $PM_{2.5}$ dataset; the Exclude Omkoi analysis excluded participants from Omkoi; and the NTAQHI-only analysis was restricted to directly measured NTAQHI $PM_{2.5}$ concentrations.

Table S8. Adjusted $PM_{2.5}$–LTL associations per IQR increase in exposure, with wild cluster bootstrap inference.

Exposure window	IQR (μg/m³)	Adjusted B per IQR increase	Wild cluster bootstrap p-value
0–7 days	4.63	−0.005	0.787
0–14 days	6.80	−0.008	0.554
0–30 days	8.13	0.053	0.342
0–60 days	7.25	0.079	0.247
0–90 days	6.25	0.092	0.321

Note: Bootstrap p-values were obtained using Webb weights and 99,999 replications, with clustering defined by the eight site–date groups. These p-values were not adjusted for multiple comparisons.

Table S9. Regression diagnostics for the five $PM_{2.5}$ exposure-window models of relative leukocyte telomere length.

Diagnostic	0–7 days	0–14 days	0–30 days	0–60 days	0–90 days
Participants, n	179	179	179	179	179
Breusch–Pagan test, p-value	0.999	0.992	0.950	0.986	0.995
Shapiro–Wilk test of residuals, p-value	0.037	0.029	0.097	0.077	0.059
Minimum studentized residual	−2.79	−2.77	−2.70	−2.62	−2.50
Maximum studentized residual	3.19	3.24	3.23	3.31	3.33
Maximum Cook's distance	0.095	0.082	0.095	0.100	0.092
Maximum leverage	0.191	0.188	0.172	0.165	0.162

Note: Diagnostics refer to the covariate-adjusted linear regression models reported in Table 3
